# Changing Metabolism in Differentiating Cardiac Progenitor Cells—Can Stem Cells Become Metabolically Flexible Cardiomyocytes?

**DOI:** 10.3389/fcvm.2018.00119

**Published:** 2018-09-19

**Authors:** Sophia Malandraki-Miller, Colleen A. Lopez, Heba Al-Siddiqi, Carolyn A. Carr

**Affiliations:** Department of Physiology, Anatomy and Genetics, University of Oxford, Oxford, United Kingdom

**Keywords:** heart, progenitor cells, substrate metabolism, mitochondria, differentiation, cardiomyocytes

## Abstract

The heart is a metabolic omnivore and the adult heart selects the substrate best suited for each circumstance, with fatty acid oxidation preferred in order to fulfill the high energy demand of the contracting myocardium. The fetal heart exists in an hypoxic environment and obtains the bulk of its energy via glycolysis. After birth, the “fetal switch” to oxidative metabolism of glucose and fatty acids has been linked to the loss of the regenerative phenotype. Various stem cell types have been used in differentiation studies, but most are cultured in high glucose media. This does not change in the majority of cardiac differentiation protocols. Despite the fact that metabolic state affects marker expression and cellular function and activity, the substrate composition is currently being overlooked. In this review we discuss changes in cardiac metabolism during development, the various protocols used to differentiate progenitor cells to cardiomyocytes, what is known about stem cell metabolism and how consideration of metabolism can contribute toward maturation of stem cell-derived cardiomyocytes.

## Stem cell therapy for the heart

**Figure 1:**
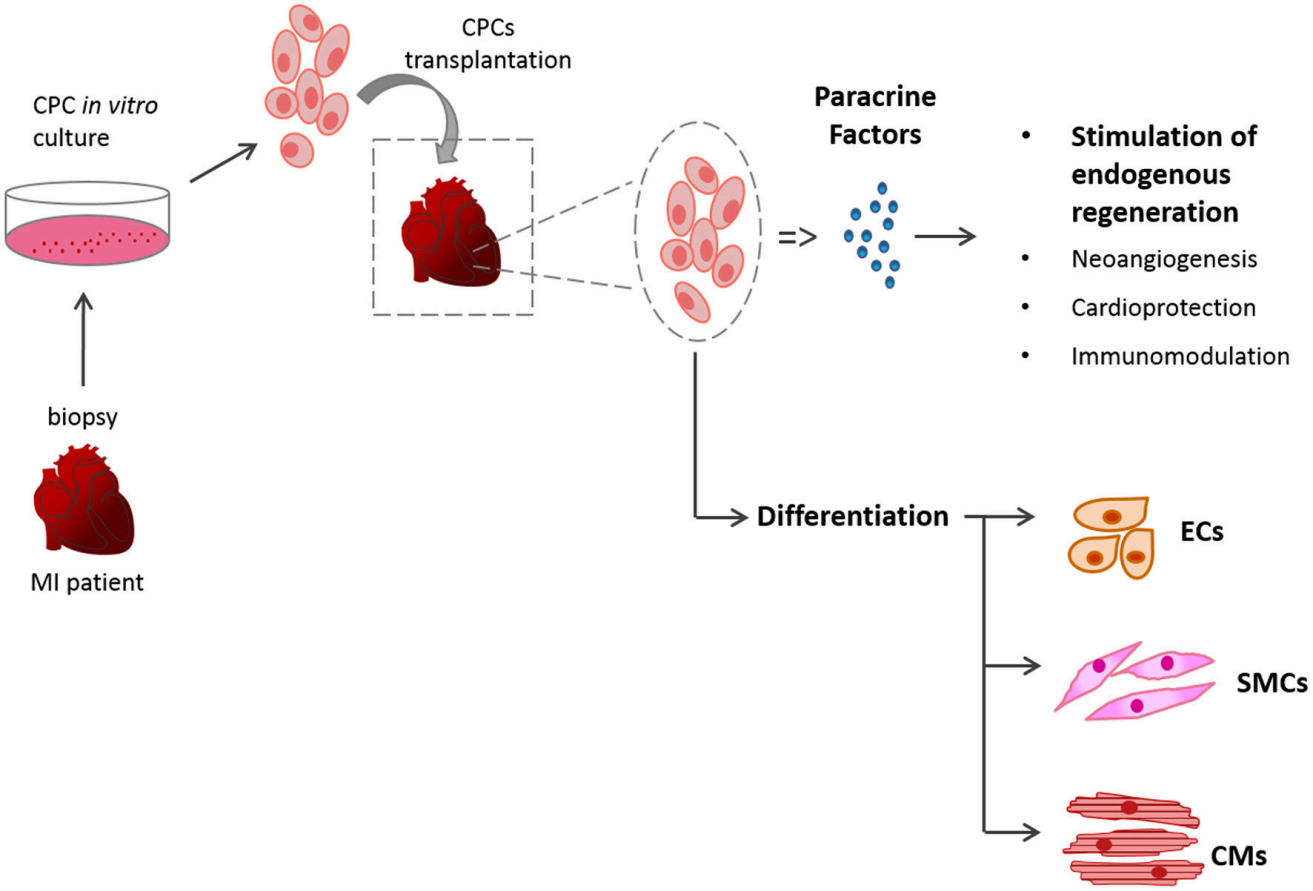
Schematic of SCT. The mechanisms of action of the transplanted cardiac stem cells (CSCs) can be by differentiation of the donor cells or via paracrine mechanisms.

Myocardial infarction (MI) is the primary cause of disease-related death in the world with no reliable therapy (1). Acute coronary syndromes, like MI, account for half of all cardiovascular deaths in the industrialized world, with around 20% of patients developing heart failure (HF) and having a 1-year mortality rate (2, 3). Current therapeutic strategies focus on reperfusion, thrombolysis and reducing the workload of the heart using pharmacological agents or surgical procedures (4–6). Recent advances in treatment have improved time to reperfusion, but progress in identifying efficient therapies to offer more than symptom alleviation and support the surviving myocardium is yet to result in substantial clinical benefit (7, 8). The only current long-term solution is heart transplantation but with the limited numbers of donors, and the need for chronic immunosuppressants (9), the search to find an alternative solution to the problem of end stage HF is becoming increasingly urgent. MI can lead to a loss of up to 1 billion cardiomyocytes, which cannot be replaced due to the insufficient degree of regeneration in the adult heart (10). Although the heart is no longer considered a post-mitotic organ, the turnover of cardiomyocytes in the adult heart is around 1% per year (11) which is insufficient to counter the loss caused by MI. Stem cell therapy (SCT) has the potential to regenerate the damaged tissue and restore its contractility, harnessing the self-renewal and differentiation potential of stem cells (SCs) (12). *In vivo*, the transplanted cells can act *via* a combination of the following mechanisms; (a) replicate themselves and/or differentiate to mature cardiomyocytes; (b) stimulate the endogenous cardiac cells to regenerate; (c) exert a beneficial effect via paracrine mechanisms of action (13) (Figure 1).

## Types of stem cells for therapy

A wide range of cells have been tested both in animal models or early-stage human clinical trials in order to find the appropriate source for SCT (14, 15). These include bone-marrow derived cells (16–18), cardiac stem or progenitor cells (19–25), human embryonic stem cell-derived cardiomyocytes (26–29) and human inducible-pluripotent stem cell-derived cardiomyocytes (30, 31).

Bone marrow-derived stem cells were claimed to differentiate into cardiomyocytes that spontaneously beat after 2 weeks in culture (17) or into myotubules that, when injected into infarcted hearts, stimulated angiogenesis and generated cardiac-like cells (16). In addition, it was reported that when bone marrow-derived stem cell growth factor receptor-positive/linage negative (c-kit+/lin-) cells were injected into infarcted tissue, they generated new cardiac cells and blood vessels and re-muscularised the damaged region (18). However, later studies showed that bone marrow-derived cells do not trans-differentiate into cardiomyocytes and that retained transplanted cells adopted a mature haematopoetic fate (32, 33). Bone-marrow derived mesenchymal cells have also been shown to improve cardiac function following MI, although repair is now thought to result from the delivery of a cocktail of beneficial cytokines which induce angiogenesis, limit scar fibrosis and may activate endogenous cardiac progenitors (34–36). Other key types of mesenchymal stem cells (MSCs) such as umbilical cord MSCs (37, 38), adipose-derived MSCs (39–41) and amniotic fluid MSCs (42), chosen for their ease of isolation and differentiation, have also been tested for therapeutic potential after infarction. As with bone marrow cells, any beneficial effect was deemed to be paracrine.

In 2003, a population of cardiac progenitor cells called stem cell growth factor receptor-positive (c-kit+) cells were identified (19). *In vitro*, these proliferative cells can self-renew and differentiate into myocytes. When injected into infarcted hearts, c-kit+ cells were shown to differentiate into cardiomyocytes resulting in myocardium regeneration and improved heart function (19). Subsequent studies supported the beneficial effect after cardiac injury, but suggested that the c-kit+ cells were of bone marrow origin (43) or were mast cells (44), endothelial cells (45) or maybe a mixed population of both (46). Studies looking at the issue from a developmental perspective suggested that c-kit+ cells contribute to new cardiomyocytes after injury in the neonate, but they were unable to do so in the adult heart (47), in line with previous observations regarding the role of c-kit+ cells in the neonatal heart (48). In 2011, the Anversa group used c-kit+ cardiac progenitor cells (CPCs) in a phase-I Stem Cell Infusion in Patients with Ischemic cardiomyopathy (SCIPIO) clinical trial showing encouraging results for post-MI treatment (49). However, in 2014 the results were questioned for their integrity (50). In 2013, Ellison et al. showed that c-kit+ CPCs were necessary and sufficient for cardiac recovery in rodent models of diffuse myocardial damage causing acute heart failure (51). This was subsequently challenged by studies using c-kit+ lineage tracing mouse models, and reporter lines, where it was shown that cardiac c-kit+ cells contribute to cardiomyocytes only minimally, but mainly and substantially generate cardiac endothelial cells (52–54). The debate continues, with recent publications showing that selected c-kit+ cardiac cells contain a low level (about 1%) of clonal cells that can be expanded and differentiated into spontaneously beating cardiomyocytes (55) and that c-kit expression can be identified on both CPCs and a subpopulation of cardiomyocytes and is upregulated in response to pathological stress (56).

Also in 2003, another population of cardiac progenitor cells was identified, the stem cell antigen 1 (sca-1+) cells, in the mouse heart, having stem-like self-renewal characteristics and the ability to home to the injured myocardium (20). Later it was shown that this cell population led to increased ejection fraction and neoangiogenesis, after injection into the acutely infarcted mouse heart (57). It was also shown that sca-1+ CPCs contribute to the generation of cardiomyocytes during normal aging and after injury sca-1+ cells were induced to differentiate to three cardiac cell types (44). In line with these observations, a genetic deletion of sca-1 caused primary cardiac defects in heart contractility, an impaired damage response and reduced CPC proliferation (58). Although the human sca-1 isoform does not exist, a sca-1-like cell population has been isolated from the human heart using the murine antibody and has been extensively studied by the Goumans group (59). In a similar manner to the isolation of a clonal c-kit+ population, clonal cells have been identified within selected sca-1+, PDGRFα+ mouse cells and have been shown to improve cardiac function after MI, but again by a largely paracrine mechanism (60).

In addition, a population of progenitor cells were derived, via the formation of cardiospheres, from cells migrating from adult human and murine heart explants, called cardiosphere-derived cells (CDCs) (21). It was reported that human CDCs have the ability to self-renew in culture and express the endothelial kinase insert domain receptor (KDR) and other known stem cell markers (CD-31, CD-34, c-kit, and sca-1) (21). This heterogeneous cell population has also been shown to have beneficial effects, both in animal models (22, 61) and in a clinical trial (62), but again via the release of paracrine factors.

CPCs have been also identified by markers of embryonic origin, like Insulin gene enhancer protein 1 (Isl1) and NK2 homeobox 5 (Nkx2.5). Isl1 is a cardiac transcription factor expressed in second heart field progenitors and cardiac neural crest cells, involved in cardiovascular development, and leading to severely deformed hearts in rodents after genetic deletion (24, 63). Nkx2.5, a homeobox-containing transcription factor, has been identified via its involvement in cardiac looping (64, 65). Isl1+ and Nkx2.5+ CPCs have been shown to differentiate into major cardiac lineages, mainly contributing to proepicardium during development (25).

Human embryonic stem cells (hESCs) are derived from the inner cell mass (ICM) of blastocysts from donated fertilized eggs (66). Human ESCs have the ability to continuously proliferate in an undifferentiated state and, when given the appropriate signals, will differentiate into any cell type. An interesting tool for cell therapy that originated from this field, are the *in vitro*–generated, stem cell–derived cardiomyocytes (SC-CMs). These have been shown to integrate structurally and functionally with healthy host cardiac tissue *in vivo* in various studies (26, 67, 68). These cells show great promise, but there are ethical concerns using hESCs in the clinic and the risk of teratoma formation (69). In 2007, Yamanaka's group were the first to report the reprogramming of human somatic cells into induced pluripotent stem cells (iPSCs), by overexpression of the transcription factors: Oct4, Sox2, KLF4, and c-myc (70). The reprogrammed hiPSCs resembled hESCs and had the ability to self-renew while maintaining pluripotency (70). Human iPSCs can be produced from patient-specific somatic cells, therefore overcoming the problem of immune rejection and the ethical concerns of using hESCs (69). hiPSCs have been shown to improve cardiac function, albeit with limited donor cell retention (30, 31) and used extensively as *in vitro* human-cell-based models to study basic biology and development (71), to model diseases (72) and to screen for drugs (73, 74). This is particularly important for the heart, since adult cardiomyocytes do not survive *in vitro*, as morphological and functional changes occur in long-term culture and so there has been no easy way to determine whether the effect of genetic mutations or of drug compounds that were observed in animal models would also be seen in a human cardiomyocyte. However, despite the promising *in vivo* results, the initiation of beating in SC-derived cardiomyocytes does not mean that these cells have the maturity or metabolic characteristics of mature cardiomyocytes found in the healthy heart (75). Studies have shown that SC-derived cardiomyocytes have immature calcium handling (76) and a response to drugs more akin to cardiomyocytes from the failing heart (77).

The effect of the transplantation environment on enhancing the maturation of human pluripotent SC-derived cardiomyocytes has been studied in rats. Despite their capacity to survive and form grafts, they failed to improve adverse remodeling or overall cardiac function after chronic MI (28). Approaches to enhance their efficacy, via preconditioning the cells and host environment, are currently being investigated [reviewed here (78)].

## Cardiac metabolism

The heart is a fascinating organ that beats 100,000 times a day and pumps 7,200 L of blood through the body, in the same period using 35 L of O_2_ for energy production. It requires about 6 kg of adenosine triphosphate (ATP), which it utilizes at a rate of 30 mg per second to sustain myocardial contraction and maintain ion homeostasis (79, 80). Since the heart has a low capacity for energy storage (81), an array of metabolic networks guides ATP production rates, based on demand. The heart has been characterized as a metabolic omnivore, being able to use a variety of substrates for energy production [see reviews (82, 83)]. Glucose, pyruvate, triglycerides, glycogen, lactate, ketone bodies, fatty acids (FAs) of different chain-lengths and certain amino acids are among the energy-providing substrates of the heart. It is responsible for almost 10% of the whole body fuel consumption; with FAs accounting for 70% of ATP production and carbohydrates for the remaining 30%.

**Figure 2:**
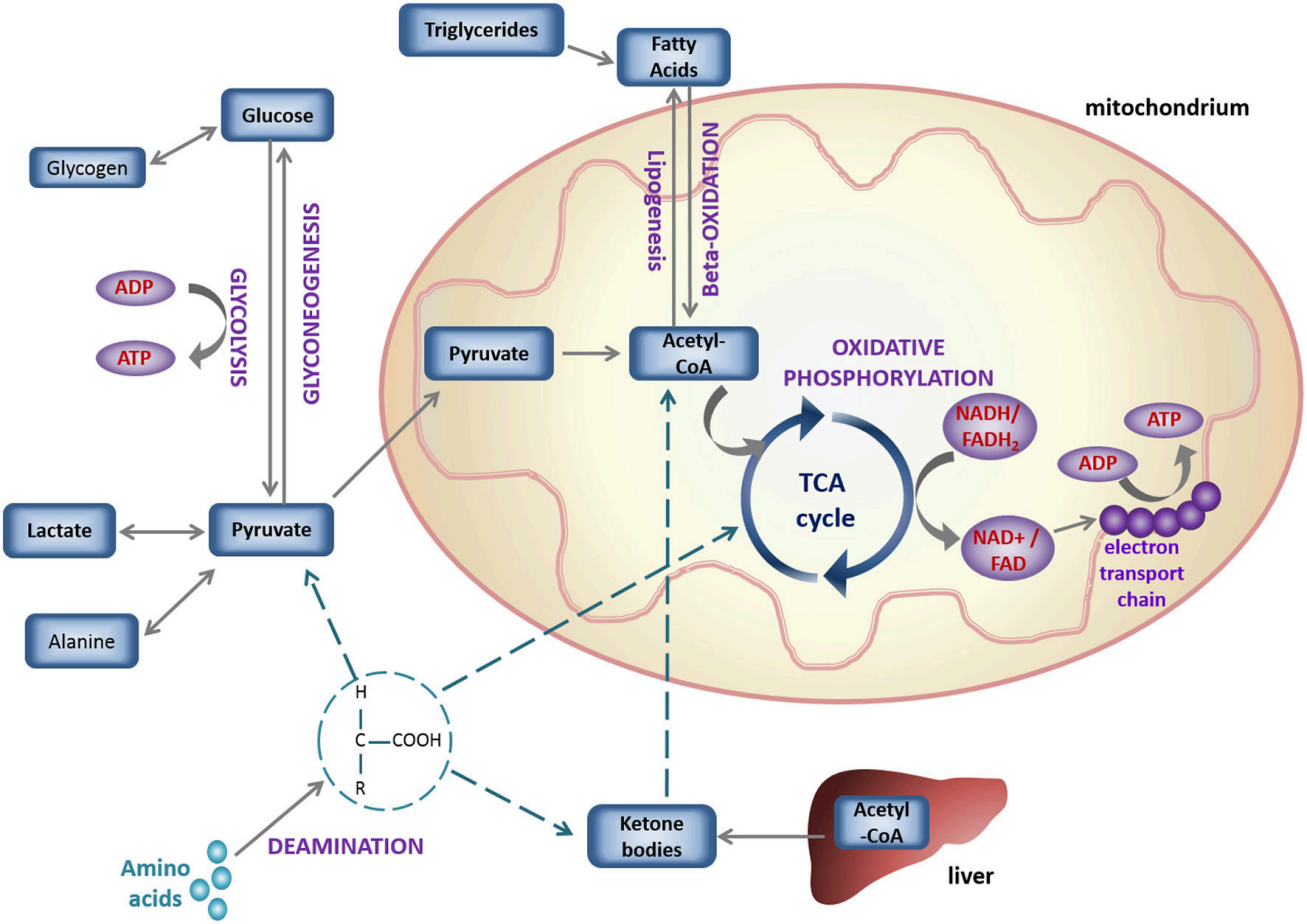
Schematic overview of basic cell metabolic pathways for energy production.

Energy, in the form of ATP, can be produced in the cytosol via glycolysis (Figure 2); catabolism of glucose derived from carbohydrates. The end-product of glycolysis is pyruvate, which can be further reduced to produce lactate. In case of carbohydrate shortage, gluconeogenesis of pyruvate, re-oxygenation of lactate or glycerol metabolism, can be used as sources of glucose synthesis (82, 84). Alternatively, pyruvate can enter the mitochondria in the form of acetyl-coenzyme A (Acetyl-CoA) and be oxidized in the TCA cycle (also known as the Krebs cycle), in a process called oxidative phosphorylation (85). The reducing equivalents of this chained reaction act as hydrogen carriers (Nicotinamide Adenine Dinucleotide Hydrogen; NADH and Flavin Adenine Dinucleotide Hydrogen; FADH_2_) and enter the electron transport chain (ETC). There the coupled-transfer of electrons and H^+^ creates an electrochemical proton gradient that leads to the production of ATP.

ATP can also be generated by the degradation of lipids (including triglycerides) into FAs, which are metabolized in the mitochondria via beta-oxidation (Figure 2), which converts Fatty Acyl-CoA to Acetyl-CoA for flux into the TCA cycle and ATP synthesis as above (86). Under aerobic conditions, more than 95% of ATP production comes from oxidative phosphorylation (86) and in the healthy heart 50–80% of the energy is generated via beta-oxidation (87). Oxidative phosphorylation yields 36 ATP/glucose molecule, being more efficient than glycolysis (2 ATP/glucose). Lipids, due to their reduced state are more oxygen-demanding than glucose (producing 2.8 ATP/O_2_, vs. 3.7 ATP/O_2_), but they are more energy-dense with a much higher yield of ATP/carbon (depending on the chain length of the parent FA) (79, 88).

The heart has a remarkable ability to adapt to changes in its physiological state by selecting the most efficient substrate, depending on the conditions of its environment (82). For example, as FAs require more oxygen than glucose, to generate the same amount of ATP (82, 89), upregulation of the hypoxia-inducible factor (HIF) under hypoxia has been shown to increase glycolysis and suppress mitochondrial oxidative metabolism (83, 90), shifting toward the more oxygen-efficient fuel: glucose. A network of interrelated signaling pathways control the flux of glucose and fatty acid metabolism to enable the heart to switch substrates rapidly. This was first described as the glucose-fatty acid cycle by Randle in 1963 (91), but the complexity of this network is yet to be fully explored (92). Under conditions of starvation, acetyl-CoA in the liver can form ketone bodies, that are water-soluble forms of this metabolite, and can be efficiently used by the brain and other oxidative organs (82) (Figure 2).

### Cardiac metabolism during development—metabolic switch (1)

**Figure 3:**
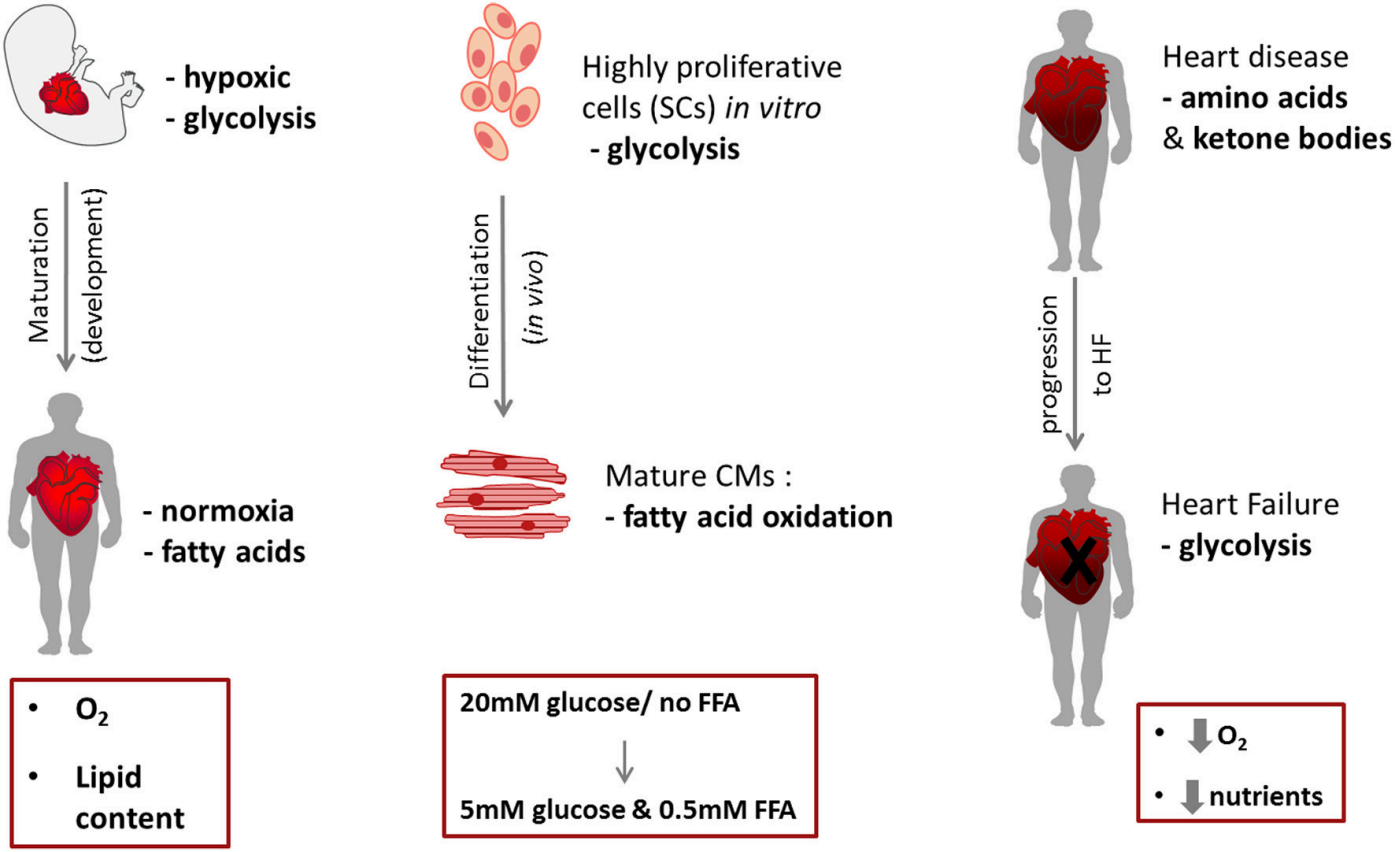
Schematic of metabolic switches during cardiac development (left) and cell differentiation (right).

The fetal heart is adapted to an environment of low oxygen and low fatty acid content, so fetal cardiomyocytes are highly dependent on glycolysis for ATP production (93). In addition, lactate availability allows for energy production via lactate oxidation. During development, the heart undergoes a major metabolic alteration; the main physiological changes during the transition to the post-natal stage are the increased workload, and the demand for growth, that cannot be met by glucose and lactate consumption alone (94, 95). Interestingly, immediately after birth some studies suggest that the main energy biogenesis mechanism is still glycolysis (96). The post-natal increase in both circulating levels of free FAs (due to dietary alteration and lipid content in maternal milk) and in O_2_ levels mediates a switch from glycolysis-dependence, to predominantly relying on oxidative metabolism as mature cardiomyocytes (93, 97) (Figure 3). After birth, the “fetal switch” to oxidative metabolism of glucose and fatty acids has been linked to the loss of the regenerative phenotype (98). Neonatal mouse hearts can regenerate in the first postnatal week but this is lost after day 7 (99). Puente et al. investigated exposure of neonatal mice to hyperoxia (100% oxygen) or mild hypoxia (15% oxygen) and found that hyperoxia induced cardiomyocyte cell cycle arrest after birth whereas hypoxia prolonged the regenerative window (98). They hypothesized that this effect resulted from increased oxidative stress that accompanied the induction of oxidative metabolism and showed that treatment with an antioxidant resulted in a significant increase in cardiomyocyte mitosis in the first weeks after birth. During the early postnatal period, as cardiac energy demands increase, the number of mitochondria in cardiomyocytes increases dramatically (100, 101). A recent study has shown that the HIF1 signaling localisation pattern controls the embryonic switch toward oxidative metabolism, disruption of which affects cardiac maturation. The cardiac compartment where HIF was absent, the trabeculae, has increased oxidative metabolism, as well as higher mitochondrial content (102). Several studies demonstrated an increase in PPAR coactivator 1α (PGC-1), as well as Peroxisome Proliferator-Activated Receptor α (PPARα), mRNA levels in mice or rats during development (103, 104). The regulatory mechanism involves genes encoding several key mitochondrial ETC proteins, specifically the transcription factors nuclear respiratory factors-1 and−2 (NRF-1 and−2) (103, 105). Disruption of the ETC function, during cardiac development, leads to disrupted mitochondrial organization in the cardiomyocytes, resulting in perturbed sarcomere formation and contraction (106). Various mouse models have revealed that disruption in mtDNA processes leads to embryonic lethality (107). In addition, signaling pathways involving the Nuclear factor of activated T-cell (NFAT) family of transcription factors play a role in mitochondrial biogenesis and cardiac function. Mice with nfatc3 and nfatc4 deletion had abnormal mitochondrial structure and reduced oxidative capacity, eventually dying after E10.5 (108). The ERRα/ERRγ axis plays a crucial role in mitochondrial energy production, as well, and their deletion leads to premature lethality in mice (109). Disruption of the ERRγ gene leads to impaired mitochondrial function, eventually blocking the switch from glycolysis to oxidative phosphorylation and leading to early prenatal death in mice (110).

### Cardiac metabolism in MI and HF—metabolic switch (2)

Following MI, tissue ischaemia leads to hypoxia, which in turn activates long-term HIF signaling in the myocardium (111). During MI, the ischemic region relies solely on glycogen, as an anaerobic fuel for energy production (112). The absence of oxygen for OXPHOS means that NADH and FADH2 are accumulated, affecting fatty acid enzyme reactions and leading to a build-up of fatty acid intermediates and resulting in disruption of mitochondrial cristae and function (87). Reduction in fatty acid oxidation upregulates anaerobic glycolysis and lactate production leading to intracellular acidosis (87). HIF1α activation upregulates the transcription of Bcl-2 adenovirus E1B 19 kDa-interacting protein 3 (BNIP3), which triggers selective mitochondrial autophagy (113, 114). Under normal conditions, BNIP3 expression is suppressed by the nuclear factor kappa beta (NF-κB) pathway (114). In addition, HIF1α upregulates the transcription of pyruvate dehydrogenase kinase 1 (PDK1), which inactivates pyruvate dehydrogenase, thereby blocking the entry of pyruvate into the mitochondria for oxidative phosphorylation (113). These HIF-1 mediated changes play key roles in reducing mitochondrial oxidative metabolism and thereby in reducing the generation of reactive oxygen species (ROS) (115). Following reperfusion, oxidative metabolism is restored but the resulting induction of ROS can lead to mitochondrial damage (116). As the heart progresses to failure, flux from pyruvate into the TCA cycle decreases (117). Mitochondria can be found with membrane and ETC defects (118, 119), as well as reduced respiratory capacity (119, 120) and reduced oxidative phosphorylation (121–123). These findings are consistent with the concept that, during HF, the metabolic state of the heart resembles that of the fetal stage, switching to glycolysis rather than mitochondrial oxidative metabolism (124); with increased glucose uptake (125) and glycolysis (86) and either no change or a decrease in glucose oxidation (125, 126).

The aforementioned perturbations in cardiac metabolism lead to a dangerous environment, due to the excess of lactate and oxidative metabolic intermediates. Progressive heart failure induces, at a cellular level, increased conversion to lactate, increasing cell acidosis (127). However, amino acids such as aspartate play a role in reducing damage that occurs as a result of lactate accumulation via upregulating the conversion of pyruvate to alanine. Glutamate and aspartate improve the production of ATP as they produce intermediates that feed the TCA cycle for further oxidation (128). Studies have reported the reduced availability of amino acids in heart failure patients, leading to the depletion of some valuable amino acid derivatives that are important for normal cardiac function (l-carnitine and creatine) (129). However, there is also evidence of their accumulation during HF, as seen in a study on failing mouse hearts where genes associated with amino acid catabolism were downregulated during compensated hypertrophy and overt failure (130). Transcriptomic analyses reflected downregulation of genes involved in amino acid degradation pathways [proline, alanine, tryptophan, and mainly branched-chain amino acids (BCAAs)] (131–133). Using a genetic mouse model, Sun et al. demonstrated that a deficiency in BCAA catabolism induced heart failure under mechanical overload, resulting from increased oxidative stress (132). Similarly, Li et al. found that whilst chronic accumulation of BCAAs did not affect cardiac energetics and function in the healthy mouse heart, glucose oxidation was decreased which increased ischaemic injury after myocardial infarction (134).

The importance of mitochondrial integrity for maintenance of cardiac function has been also highlighted through several conditions which are characterized by mitochondrial mutations or abnormalities, causing among others: cardiomyopathy, neuromuscular dysfunction, diabetes mellitus and even sudden death (122, 135). In addition, the efficiency of the ETC is found to decline with age, which in turn decreases ATP generation (135, 136).

### Cardiac metabolism from stem cells to adult cells and vice versa—metabolic switch (3)

Metabolic changes during embryonic development have been reviewed in detail by Johnson et al. (137). Mammalian oocytes contain low levels of glycogen and fat but have substantial stores of amino acids and protein which have been estimated to be sufficient for the energetic needs of the first few days of development (137). The preimplantation embryo relies on the mitochondria inherited from the oocyte and on pyruvate oxidation for ATP production (138, 139). There is a gradual switch in metabolism during the preimplantation stage (morula, blastocyte) from aerobic oxidation to anaerobic glycolysis in preparation of the low-oxygen environment in the uterine wall (137). As the preimplantation embryo undergoes cell division there is a reduction in mitochondrial DNA copy number (mtDNA) and mitochondrial density (138) and an upregulation of glucose transporter 3 and hexokinase gene expression until anaerobic glycolysis increases to maximal rates as the blastocyst implants into the uterus (137). ESCs originate from the inner cell mass of preimplantation blastocyst, therefore, these cells show high rates of glycolysis and low oxidative phosphorylation to support the rapid cell proliferation (138, 140). Similarly, ESCs derived from *in vitro* cultured ICMs (141) or ESCs cultured *in vitro* (142) show high rates of glycolysis and low oxidative phosphorylation. A comparison of the metabolomics and energetics of iPSCs with those of ESCs showed many similarities and confirmed that, during reprogramming, somatic cells convert from an oxidative state to a glycolytic state in pluripotency (143).

The reprogramming factors, c-Myc and Lin28, have been shown to enhance glycolysis and nuclear reprogramming (140) and reprogrammed pluripotent stem cells show upregulation of glycolytic enzymes such as the glucose transporter GLUT1, hexokinase (HK), phosphofructokinase (PFK) and LDH (144). In addition, hiPSCs have increased levels of intracellular glucose-6-phosphate, which feeds into the pentose phosphate pathway (PPP) to generate the reducing factor NADPH, which is used for biosynthesis of FAs and nucleotides necessary for rapid proliferation (144). Stimulation of glycolysis with D-fructose-6-phosphate or by PDK1 activation has been shown to enhance the efficiency of iPSC reprogramming (145). Mitochondrial morphology changes from being elongated and tubular-shaped with well-developed electron-dense cristae in somatic cells, to being spherical with small-undeveloped cristae in hiPSCs, with a transition from an extensive cytoplasmic networks in somatic cells, to a predominantly peri-nuclear location in hiPSCs (146). Prieto et al. found that this fragmentation was not associated with mitophagy but was induced by activation of dynamin-related protein 1 (Drp1), which fragments mitochondria in a GTPase-dependent manner (147). They showed that Erk activation in early reprogramming induced Drp1 phosphorylation which was critical to the reprogramming pathway. Studies in human and mouse ESCs have also found a small number of rounded and immature mitochondria with under-developed cristae (106, 148). Moreover, multipotent stem/progenitor cells show the same characteristics; for example, mitochondria in HSCs are relatively inactive and ATP content increases in lineage-committed progenitors compared with HSCs (149).

Due to the ongoing controversy as to whether the heart contains resident progenitor cells (150), little is known about the metabolism of cardiac stem cells in the adult heart. However most adult endogenous progenitor/stem cells reside in hypoxic niches (151), in a quiescent state, and therefore the metabolic rate is presumed to be low. CPCs isolated from the heart, by marker selection, tissue digestion or from explants, and expanded *in vitro*, are proliferative and grown in culture medium containing high levels of glucose and little or no FAs. Basal cellular homeostasis involves processes like protein turnover, DNA repair, and vesicle trafficking and therefore proliferating cells, in addition to homeostasis maintenance, need energy for anabolic processes such as cell division and growth. Proliferative cells, such as cancer cells, have been suggested to predominantly rely on glycolysis for ATP production, irrespective of oxygen presence; this metabolic paradox, first observed by Warburg in 1956 (152) has been termed “aerobic glycolysis.” Apart from Warburg's observations on cancer cells, mouse fibroblasts (153) and human (154) and mouse (155) lymphocytes have been shown to utilize “aerobic glycolysis,” when stimulated to proliferate. Various studies have supported the conclusion that the major function of aerobic glycolysis is to supply glycolytic intermediates for anabolic reactions in cells, thus being the metabolic pathway of choice during cell proliferation [for Review see (156)]. Although cells in high glucose medium predominantly metabolise glucose, they can use other substrates if provided. Human bone marrow MSCs have an active oxidative metabolism with a range of substrates and can produce more ATP from substrate oxidation than glycolysis with certain substrates (157). For example, the ketone body, acetoacetate, can be oxidized at up to 35 times the rate of glucose. Many proliferating mammalian cells, such as human MSCs, also consume glutamine to provide material for biosynthesis (158). Glutamine, as a carbon source, can supply the TCA cycle with intermediates that can be used for the production of new macromolecules in cells. A recent study by Hosios et al., in 2016, argued that glutamine contributes most to protein synthesis, suggesting that anaplerosis of glutamine in the TCA cycle is serving mainly amino acid biosynthesis (159).

When stem/progenitor cells differentiate to cardiomyocytes they need to increase the number of their mitochondria and upregulate FA metabolism (Figure 3). A study comparing hiPSC-derived cardiomyocytes (hiPSC-CM) to hiPSCs, showed an increase in mitochondrial relative abundance and activity (mitochondria membrane potential) as a result of cardiac differentiation (160) and that additional mitochondrial maturation in hiPSC-CMs could be achieved by long-term culture (3 months). Comparison of substrate metabolism in ESCs with that in ESC-derived cardiomyocytes has shown increased oxidative metabolism after differentiation (161) and that the respiratory capacity of cardiomyocytes was higher than in ESCs, resulting in an increased ADP:ATP ratio in the cardiomyocytes (106). The mitochondria become elongated with abundant and organized cristae in cardiomyocytes and formed into networks which filled the cytoplasm (106).

## Why study stem cell metabolism

The metabolism of any cell type *in vitro* depends on energy requirements and substrate availability. Cardiac progenitor cells, when expanded *in vitro*, reside in a high glucose environment and rely on aerobic glycolysis for energy generation. CPCs will experience a shift in substrate availability following transplantation *in vivo* (Figure 3), being transferred from the culture medium, which contains about 5–25 mM glucose (depending on the culture protocol) and no FFA, to substrates in plasma that vary substantially. Glucose levels in mice have been measured at between ~3.4 and 9.6 mM (2.8–7.5 mM in rats) (162, 163) and ~0.18–0.6 mM FFA (164). In humans, healthy plasma glucose levels are around 5 mM and FFA 0.5 mM (165). This alteration is bound to cause changes in the metabolic machinery of the cells which might be one of the stimuli that induce differentiation following transplantation, but may also result in increased release of ROS and cellular damage.

The various protocols used for CPC differentiation focus on pharmacological reagents and cytokines, and do not refer to or take into account the substrate composition. This is a striking fact especially knowing how metabolic changes affect the function of the cells (166) and how transition from glycolysis to FA oxidation affects cell maturation (93), and *vice-versa* (144). iPSC-CM have been shown to integrate structurally and functionally with healthy host cardiac tissue *in vivo* in various studies (26, 67, 68). Despite the promising *in vivo* results, the initiation of beating in iPSC-CM does not mean that these cells have the metabolic characteristics of mature cardiomyocytes found in the heart. Studies have shown that SC-CMs have immature calcium handling (76, 167) and a response to drugs more akin to cardiomyocytes from the failing heart (77). Despite the observed mitochondrial remodeling and upregulation of oxidative metabolism previously discussed, newly differentiated iPSC-CM in culture have been shown to retain a predominantly glycolytic metabolism (168). One of the major roles of iPSC-CM is that of a drug-testing platform and this requires the differentiated cells to acquire a fully mature phenotype (74). For example, a recent iPSC-CM study showed that the electrophysiological responsiveness of iPSC-CM was dependent on their maturation state (169).

## Assessing cell metabolism

The main approaches for the investigation of substrate metabolism include the measurement of metabolic fluxes using radio-labeled substrates and of oxygen consumption.

### Radio-labeled substrates assays

Glucose oxidation rates are commonly measured using the method of the Collins et al. (163) with cell culture media containing D-U-^14^C-glucose. Glucose oxidation results in the production of ^14^CO_2_ which is trapped for analysis using a scintillation counter. For ^3^H-FA oxidation **t**he cells are incubated in media supplemented with the radioactive FA tracer of interest. FA oxidation rates are determined by the production of ^3^H_2_O from the mitochondria (170, 171). Media aliquots contain both ^3^H_2_O and ^3^H-FA, so the ^3^H_2_O is separated via a Folch extraction (170). Glycolytic rates are determined through the conversion of ^3^H-glucose to ^3^H_2_O via enolase which converts 2-phosphoglycerate to phosphoenolpyruvate and releases ^3^H_2_O as a by-product that is collected using a Dowex mesh (Sigma, UK) anion exchange column, allowing for the ^3^H-glucose to bind to the column and ^3^H_2_O to be eluted (171).

### Oxygen consumption

The rate of mitochondrial oxygen consumption (OCR) can be measured using the XF Extracellular Flux Analyzer (Seahorse Bioscience), the Oroboros O2K or the Clark-type oxygen electrode (172, 173). Seahorse XF Analyzers measure the concentration of dissolved oxygen and pH to quantify the oxygen consumption and extracellular acidification rate in the media in multi-well plates. Four injection ports in each well allow for addition of reagents. However, the Seahorse requires cells to be adhered to the wells, multiple additions of substrates are not possible and the individual wells are not self-contained and thus cells can be affected by gases from adjacent wells (172). The Clark-type oxygen electrode is embedded in individual reaction chambers and cells are added in suspension in the respiration media (173). Unlimited reagents and substrates can be added by manual addition. This system is more automated in the Oroboros O2K which also includes optical sensors to allow for detection of fluorescent dyes so that parameters such as ATP production or mitochondrial membrane potential may be measured (172).

The OCR can be measured under baseline un-stimulated conditions in media containing the substrate of interest (such as pyruvate; palmitate; malate; or oleic acid). Addition of the ATP synthase inhibitor oligomycin provides a measure of contaminating ATP synthase activity from damaged mitochondria and of proton leak, whilst the metabolic uncoupler, carbonylcyanide-p-trifluoromethoxy-phenylhydrazone (FCCP) permits measurement of fully uncoupled or maximal respiration (173). Finally, mitochondrial inhibitors such as rotenone (complex I) and antimycin (complex III) will completely inhibit mitochondrial respiration. More exquisite interrogation of the function of mitochondrial metabolism can be performed using substrates such as glutamate which assesses the second span of the Krebs cycle, succinate which enters the electron transport chain at complex II, or β-hydroxybutyrate, a fatty acid-derived substrate which bypasses β-oxidation and enters the Krebs cycle as acetyl CoA (116, 174).

### Extracellular measurement of glucose consumption and lactate production

Lactate production can be measured using the Seahorse Flux Analyzer from the extracellular acidification rate (ECAR) of the media. Glucose and lactate levels can also be determined using enzymatic assays or the ABX Pentra 400 Chemistry Analyser (Horiba Ltd. USA). Glycolysis yields two molecules of pyruvate, which can be converted to either lactate or acetyl CoA. Therefore, the ratio of glucose consumption to lactate production can be used as an indicator of the level of utilization of pyruvate in oxidative metabolism (175).

## Cardiac differentiation *in vitro*

Various methods and strategies have been applied to develop the optimal protocol for directing *in vitro* cardiac differentiation of stem cells. *In vitro* differentiation of adult endogenous CPCs is very challenging, due to their limited plasticity. Despite a variety of differentiation studies, the ability of adult progenitors for differentiation, is still under debate (176, 177). Different approaches, utilizing various differentiation factors have been used on both pluripotent SCs and CPCs, with the main ones being; DMSO, 5-Azacytidine, Ascorbic Acid, members of the TGF-β superfamily, oxytocin, dexamethasone and retinoic acid (see Table 1).

**Table 1:** Factors and media used to differentiate adult stem cells to cardiomyocytes.

**Differentiation approaches**	**References**	**Differentiated cell type**	**Cell culture medium and differentiation factors**	**Glucose concentration**
TGF-β1 family	(178)	Human atria Sca-1^+^; clonally isolated & magnetic-sorting	5 μM 5-Aza, TGF-β1 1 ng/ml, 10^−4^ M AA, IMDM/Ham's F12 GlutaMAX, 2% serum, 1% MEM amino acids, 1% insulin–transferrin–selenium	
	(179)	Human fetal and adult atrial biopsies, Sca-1^+^ magnetic-sorting		12.5 mM
	(180)	Whole mouse heart Sca-1^+^/ CD45^−^ magnetic-sorting CDCs (Isl1^+^)		
Oxytocin	(181)	Mouse or rat whole hearts, c-kit^+^/CD45^−^/Tryp^−^ magnetic-sorting	DMEM 100 nM oxytocin acetate, 50 μg/ml AA, 2% serum, 1 μM dexamethasone, beta-glycerol phosphate 10 mM, TGF-β1, 5 ng/ml, BMP2 10 ng/ml, BMP4 10 ng/ml, Dkk1 150 ng/ml	5.5. mM
	(182)	Mouse whole heart Sca-1+ magnetic-sorting	IMDM, 10% serum, 100 nm oxytocin	22.5 mM
	(183)	Rat, mouse ventricles side population cells		
5-Azacytidine	(184)	Mouse bone marrow stroma MSCs	IMDM, 20% serum, 3 μmol/L 5-Aza	20 mM
	(185)	Human adipose MSCs	RPMI + 15% FCS, 1–9 μmol/L 5-Aza	9.4 mM
	(20)	Mouse whole heart Sca-1^+^ magnetic-sorting	Medium 199, 2% FBS, 3 μM 5-Aza	5.5. mM
	(186)	Human umbilical cord MSCs	LG-DMEM, 10% FBS, 10 μM 5-Aza	5.0. mM
	(187)	Human bone marrow MSCs	LG-DMEM, 20% serum, 3 μM 5-Aza	4.4 mM
	(188)	Rat bone marrowMSCs	DMEM, 10% FBS, 10 μM 5-Aza	5.0. mM
Dexamethasone	(189)	Human atrial or ventricular c-kit^+^ -sorted	F12 medium 10% serum, 10 nM dexamethasone	9 mM
	(19)	Rats c-kit^+^/ Lin^−^ magnetic-sorting	F12 medium 10% serum, 10 nM dexamethasone	9 mM
	(190)	Dog left ventricle c-kit^+^/ Lin^−^ and Sca-1^+^/ Lin^−^ magnetic-sorting	F12 medium 10% serum, 10 nM dexamethasone	9 mM
	(53)	mouse whole heart c-kit^+^	DMEM, 10% serum, 10 nM dexamethasone	9 mM

*5-Aza, 5-azacytidine; AA, Ascorbic acid; BMP, Bone morphogenic protein; Dkk1, Dickkopf-related protein 1; DMEM, Dubecco Modified Eagle Medium; MDMD, Iscove's Modified Dulbecco's Medium; LG-DMEM: low glucose DMEM; MEM, Minimum Essential Medium; RPMI, Roswell Park Memorial Institute medium; TGFβ, transforming growth factor β*.

More specifically, 5-azacytidine (5-Aza) is a demethylating agent that allows for the exposure of genes that are normally silenced, due to hypermethylation, by inhibiting DNA methyltransferase (191, 192). Several *in vitro* studies suggested that 5-Aza can induce cardiac differentiation, on different MSC types, such as human umbilical cord-derived MSCs (186) and adult human bone marrow-derived MSCs (17, 187). Other studies have demonstrated the inefficiency of 5-Aza as a cardiac differentiation agent, showing transdifferentiation to skeletal muscle cells, rather than cardiac cells (193) as well as unsuccessful differentiation of adipose-derived stem cells (ASCs) (194) and adult mouse Sca-1+ CPCs (182). Ascorbic Acid (A.A.), is an antioxidant compound which has been shown to increase the expression of cardiac genes and their proteins and to lead to beating cardiomyocytes in mouse ES cells (195). Cao et al. in 2012 demonstrated that A.A. was able to induce cardiac differentiation and maturation in several human and mouse iPSC lines (196). In contrast, treatment of BM-derived MSCs with A.A. triggered their proliferation and differentiation into osteoblasts and adipocytes (197). Another key player in cardiac differentiation is transforming growth factor-beta 1 (TGF-β1) which is thought to drive cardiac differentiation by inducing the cardiac transcription factor Nkx2.5 (198). Goumans et al. used TGF-β1 to induce differentiation of adult atrial Sca-1^+^ CPCs, in combination with 5-Aza and A.A (178, 179). The mechanism of action involves phosphorylation of Smad2, that leads to the expression of cardiac-specific proteins (179). Dexamethasone is a glucocorticoid compound with immunomodulatory properties. Initial studies showed that it stimulates differentiation and maturation of osteogenic progenitor cells (199). The osteogenic effect of dexamethasone has since been demonstrated on MSCs (200–202). Interestingly, various groups have used it as the main agent of differentiation of adult selected c-kit+ CPCs to cardiomyocytes (19, 181, 189, 190). One of the most successful *in vitro* differentiation protocols for adult cardiac progenitors uses staged treatment with oxytocin, BMP2/4, TGF-β, and DKK1 which has been shown to induce cloned c-kit+ progenitor cells to form beating cardiomyocytes (181)

Cardiac directed differentiation protocols used for pluripotent SCs can be divided into two main groups, the Embryoid Body (EB)-based method (203, 204) and the monolayer-based method (30, 205–208). These protocols are discussed briefly here but a more comprehensive review has been given by Mummery et al. (209). Although cardiac differentiation protocols vary, most monolayer-based methods involve stage-specific activation and inhibition of signaling pathways that control heart development, replicating the early cardiac developmental stages in the early embryo (mesoderm induction, mesoderm cardiac specification, and generation of cardiomyocytes). The signaling pathways that are involved in most directed cardiac differentiation protocols are Activin/Nodal and bone morphogenetic protein (BMP) signaling (203), which are members of the (TGFβ) signaling pathway and the fibroblast growth factor (FGF) (30, 203), and the Wnt signaling pathways (205–208). Activin A, BMP4, and Wnt3 induce the mesoderm, and upregulate the expression of Brachyury T, whereas inhibition of Wnt signaling at later stages of differentiation has been shown to induce mesoderm cardiac specification (209). Following these steps, cells are generally cultured with media in the absence of growth factors, to allow cardiomyocyte maturation to give spontaneous beating. The distinct effects of Wnt signaling during various stages of cardiac development has been thoroughly investigated, both *in vitro* and *in vivo* (210). More specifically, it was shown with *in vivo* gene function studies in the mouse that Wnt initially enhances mesoderm commitment, while it later hinders the induction of cardiogenesis, and that it stimulates the proliferation of Isl1+ cardiac progenitors (211, 212). In addition, a study in zebrafish embryos demonstrated the switch from the inductive to the inhibitory role, of Wnt on cardiac formation, during a short 1-hour window prior to gastrulation (213). In chick embryos, treatment with Wnt antagonists *in vivo* enhanced expression of cardiac muscle differentiation markers and increased expression of Isl1 and Nkx2.5 in splanchnic mesoderm (214). *In vitro*, Murry's lab in 2010 showed that supplementation with exogenous Wnt at the point of initiation of cardiogenic differentiation of hESC enhanced the cardiac marker expression, while the same effect was induced by antagonism of endogenous Wnts at a later stage (208).

Differentiation protocols were first developed using hESCs and then were translated to iPSCs. Table 2 summarizes and compares some of the published differentiation protocols and shows a shift from the EB-based model using hESCs to the monolayer-based model using hiPSCs, which is the commonly model used currently. Table 2 also takes note of the fact that all these protocols are differentiated in variable concentrations of glucose (4.5–25mM) and insignificantly low amounts to no fatty acids (at most 2 μM). In general, spontaneously beating EBs are generated from cells in suspension using a serum-based media to induce spontaneous cardiac differentiation (27, 204, 216). The beating areas are then hand-picked from the rest of the EB or flow-sorted. Laflamme et al., played a key role in the enhancement of cardiac differentiation protocols by shifting from the serum-induced EB-based differentiation protocol that generated 10–15% spontaneously beating EBs (218) to the serum-free monolayer-based differentiation generating >30% cardiomyocytes using Activin A and BMP4 (27). In 2011, Burridge et al. systematically compared 45 variables added to EBs formed by forced aggregation which they tested on four hESC and seven hiPSC lines (222). Their optimized method included addition of BMP4 and FGF2, with polyvinyl alcohol to aid EB formation, serum and insulin to induce oxidative metabolism and with staged exposure to physiological (5%) oxygen. The growth factor-directed differentiation enabled cells to be differentiated as monolayers, thereby introducing more straightforward, and hopefully more reproducible, methods of differentiation (209). Lian et al. also optimized the Wnt-based protocol by removing insulin during early stages of differentiation (205). They had found that when iPS cells were differentiated using a Gsk3β inhibitor, Activin A, and BMP4, the presence of insulin in the early stages had a strong inhibitory effect but that this was not seen when cells were differentiated by manipulation of Wnt signaling. They identified an interplay between insulin signaling and Wnt signaling which coordinates to influence differentiation to cardiomyocytes and therefore introduced staged removal and re-addition of insulin to their differentiation protocol (205).

**Table 2:** Factors and media used to differentiate pluripotent stem cells to cardiomyocytes.

**Different-iation method cell type**	**Growth factor or small molecules**	**Culture media**	**Glucose concent-ration**	**% of Cardiac markers**	**References**
EB-Method hESCs iPSc	Cells in suspension -optionally differentiated with serum-based media, DMSO, all-trans retinoic acid or 5-Aza	80% KO-DMEM, 1 mMol L-glutamine, 1.1-1.2mMol B-ME,1% NEAA, 20% FBS	4.5mM	8.1% spontaneously beating EBs. In beating EBs, 29.4% cTnI+ cells. 25% spontaneously beating EBs by day 8 and 70% by day 16 of differentiation 5-Aza enhanced levels of cardiac α-MHC 10 to 15% positive for sarcomeric MHC	(204) (215) (216) (217) (218)
Monolayer hESCs hiPSC	Guided differentiation: h-Activin A, h-BMP4 +/- staged addition of h-Wnt3a & h-DKK1	RPMI+B27	11.1mM Note: 2uM of oleic acid	> 30% CMs Differentiated cells underwent Percoll gradient centrifuge for CM enrichment (69 ± 10% CMs) Increase in sarcomeric MHC+ cells from 4 to 27%	(27) (70) (208)
EB-Method hESCs hiPSC	END-2 method (Insulin depletion, PGI2, p38 inhibition)	80% KO-DMEM, 2 mMol L-glutamine,10ng/ml bFGF, 1.2 mMol B-ME, 7.5% FCS	4.5mM	50% beating CM	(219) (220)
EB-Method hESCs	Guided differentiation: h-Activin A, h-BMP4, h-bFGF, h-VEGF, h-DKK1	StemPro-34 (Base) + [L-glutamine, AA, optional MTG, P/S]	>25mM	KDR+ selected cells 35 ± 6% cTNT+, enriched to 57 ± 4% by monolayer culture. DKK1 on day 4 gave 2-fold enrichment of CTNT+ cells	(221)
				KDR+/PDGFRA+ cells, 50–70% cTNT+ cells in beating EBs. 80% cTNT+ cells by monolayer culture	(203)
EB-method iPSCs	Forced aggregation EBs with guided differentiation:BMP4, FGF2, Staged O_2_ levels	RPMI (L-glutamine) 20% FBS on day 3 Insulin on days 0-2 and day 4	9–11mM Note: 2uM FA	Contracting EBs contained 64–89% of cardiac troponin I^+^cells	(222)
Monolayer hiPSCs	Guided differentiation: h-Activin A, h-BMP4, h-bFGF	StemPro-34 + [L-glutamine,MTG, AA, P/S]	>25mM	Spontaneously beating sheets of CMs 40 ± 15% CMs	(30)
Monolayer hESCs hiPSCs	Guided differentiation method: h-Activin A, h-BMP4, h-bFGF, + Matrigel or CHIR99021, IWP4/IWP2	RPMI+B27(-insulin) [d0-6 of differentiation]; RPMI+B27 [from d7 of differentiation]	11.1mM Note: 2uM oleic acid	Matrigel addition on day−2 and day 0 generated 80% cTnT+ CMs	(223)
				Spontaneously beating sheets of CMs, 87% cTNT+ CMs	(207) (206) (205)

*B-ME, beta-mercaptoethanol; CM, cardiomyocytes; MTG, monothioglycerol; P/S, penicillin/streptomycin; AA, ascorbic acid; NEAA, non-essential amino acids*.

## Media composition during/after differentiation

As shown in Tables 1, 2, the majority of differentiation protocols for CPCs to cardiomyocytes had a no lipids in the media composition whilst the glucose concentration in culture media ranged from 4.5 mM to 25 mM (Here it should be mentioned that the addition of serum at percentages ranging from 2 to 20% does not allow for an absolutely clear image of the media composition). The variability of glucose concentration is striking, especially bearing in mind that a glucose level of 25 mM *in vivo* is considered hyperglycemic and leads to loss of mitochondrial networks (224, 225).

### The role of hypoxia and of reactive oxygen species in differentiation

Stem cells *in vivo* occupy an hypoxic niche and their energy yielding metabolism is likely to be hypoxic with a high reliance on glycolysis for ATP generation (226, 227). There are conflicting reports on the relationship between hypoxia and differentiation of stem cells. Hypoxia alone can revert hESC- or iPSC-derived differentiated cells back to a stem cell-like state, by re-activation of an Oct4-promoter reporter (228). In contrast, exogenous expression of HIF has been shown to promote cardiac differentiation of ESC (229). Transient hypoxia during *in vitro* cardiac differentiation upregulated the Wnt signaling pathway with increased expression of the endogenous Wnt proteins (wnt3, wnt3a, wnt9a, and wnt11), which was lost when the cells were transferred back to normoxia. This resulted in increased expression of cardiac markers such as Isl-1 and Troponin C but decreased expression of βMHC and a failure to develop the contractile phenotype (230). To further complicate the picture, Gaber et al. found a dose-dependent increase in expression of the DNA damage marker γH2AX and of senescence in ESCs differentiated under increasing exposure to hypoxia (231). These differential reports may result from the different cell types (ESC or iPSC) and species studied. Fynes et al. found that hypoxic culture of mESCs primed the cells for differentiation and resulted in increased differentiation along the mesoderm and endodermal lineages whereas hiPSCs were pushed toward a more naïve pluripotent state by hypoxia and were then primed for ectoderm differentiation (232).

As previously discussed, the loss of regenerative potential of the heart in the first weeks of life has been attributed to DNA damage resulting from the increase in ROS that accompanies upregulation of mitochondrial metabolism (98). However, ROS have also been shown to signal differentiation to several cell types, including cardiomyocytes. Transient expression of an NADPH oxidase-like enzyme with induction of ROS during embryoid body development of ESCs enhanced cardiomyogenesis, which was shown to occur *via* PI-3-kinase regulation and to be inhibited by the addition of radical scavengers (233). Similarly, NADPH oxidase (NOX)-derived ROS induced cardiac differentiation via a p38 mitogen-activated protein kinase (MAPK)-dependent pathway (234). Inhibition of mitochondrial biogenesis using shRNA targeting of PGC-1α, in hESC differentiation to cardiomyoytes, repressed mitochondrial respiration, and beating frequency (161). Levels of ROS increased during differentiation but were repressed by knockdown of PGC-1α. However, decreasing ROS levels by differentiating cells under hypoxia decreased the rate of mitochondrial biogenesis, which was stimulated by induction of ROS (161). Interestingly, in addition to reducing the beating frequency, decreasing ROS levels increased the action potential and calcium transient amplitude, but made the cells vulnerable to metabolic stress. ES cells cultured in physiological levels of glucose (5 mmol) maintained their stemness and showed reduced levels of ROS, but failed to differentiate to fully-formed cardiomyocytes (235). This was associated with reduced levels of NOX4 and MAPK which were rescued by addition of the pro-oxidant ascorbic acid.

## Strategies to mature cells

A range of strategies have been used to mature iPS- and ESC-CMs, including time in culture (236), mechanical and electrical stimulation (237, 238), addition of small molecules (239), substrate stiffness (240), genetic approaches (241–243) and growth in 3D tissues (244–246). These are reviewed in detail elsewhere (74, 247) but very few consider the medium in which the cells are cultured.

## Changing media composition to be more physiologically relevant

There is a change in energy substrate availability and metabolism during heart development from the embryo into the adult stage. However, this change in metabolism is not mimicked in most published differentiation protocols. Maturation of ESC-CMs was induced by treatment with the thyroid hormone, tri-iodo-L-thyronine, which induced an enhancement in contractile kinetics, in rates of calcium release and reuptake and in sarcoendoplasmic reticulum ATPase expression, and a significant increase in maximal mitochondrial respiratory capacity and respiratory reserve capability (239). However, in that study the newly formed cardiomyocytes were cultured in serum-free medium with no fatty acids. Similarly, where maturation of ESC-CMs was induced using members of the Let-7 family of microRNAs, increasing cell size, sarcomere length, force of contraction, and respiratory capacity, no fatty acids were added to the serum-free culture medium (243).

A few recent studies have begun to change media composition to induce a particular disease phenotype. Kim et al. used a lipogenic medium comprising insulin, dexamethasone and 3-isobutyl-1-methylxanthine to induce fatty acid metabolism in iPSC-CM derived from patients with arrhythmogenic right ventricular dysplasia. They saw a mild increase in lipogenesis with minimal apoptosis after 4–5 weeks of treatment and increased expression of PPARα. Cell maturation revealed that metabolic derangement was implicated in the onset of arrhythmia (168). Drawnell et al. induced a diabetic phenotype by treating differentiated iPSC-CM with a maturation medium containing insulin and fatty acids, but no glucose for three days, followed by treatment with a diabetic milieu of glucose (10 mM), endothelin-1 (10 nM), and cortisol (1 μM) (248). This induced an increase in sarcomere length, and in velocity and duration of action potential, in addition to increases in the myosin light chain genes, MYL2, MYL3 and MYL4; of genes involved in regulation of sarcoplasmic reticulum calcium content and an associated repression of fetally enriched genes. Correia et al. reported that shifting hPSC-CMs from glucose-containing to galactose- and fatty acid-containing medium induced fast maturation into adult-like cardiomyocytes with higher oxidative metabolism, enhanced contractility and more physiological action potential kinetics (249). Perhaps the most comprehensive investigation into the effects of changing media is that of Rana et al., who modified a basal medium with combinations of high or low glucose with galactose or fatty acids (250). They found that exclusion of glucose from the medium was needed to induce iPSC-CM to switch from glycolysis to OXPHOS for their ATP production.

## Tissue engineering approaches

It is hypothesized that culturing cells in 3D would better mimic conditions *in vivo*, such that differentiated cells would adopt a more mature phenotype. There are four main types of tissue engineering strategies that have been developed to construct contractile heart muscle equivalents: stacking monolayer cell sheets to form multi-layered heart muscle (251), cells seeded onto decellularized native tissue, cells seeded onto synthetic or biologic scaffolds (252–257) and entrapment of cells in naturally occurring biogels or hydrogels (258–260). Bian et al. cultured neonatal rat cardiomyocytes in a 3D environment and after 3 weeks saw aligned, electromechanically coupled cardiomyocytes with capillary-like structures, improved calcium handling properties. The cells had action potentials which showed enhanced conduction velocities and directional dependence on the local cardiomyocytes orientation (261). Cardiac muscle strips which were fabricated from hESC-CMs and stromal cells in collagen-based biomaterials, showed higher passive and active twitch force, aligned sarcomeres, regularly dispersed connexin-43 and N-cadherin and increased expression of maturation markers (262).

Scaffolds can be formed from synthetic polymers or from natural materials such as the cardiac extracellular matrix (ECM). Besides its structural role of giving support to surrounding cells, the ECM also has important signaling roles in cardiac development and remodeling. In recent years, it has been shown to help regulate cell survival, proliferation, migration and differentiation, for example by modulating the activity, bioavailabilty or presentation of growth factors to cell surface receptors (263, 264). ECM macromolecular proteins such as collagen (259, 265, 266), elastin (267), fibrin (268, 269) and glycoaminoglycans (270, 271) can be extracted from tissue and integrated in 3D scaffolds (267, 271) or hydrogels (268) to explore further the retention and maturation of SCs seeded. Material properties are more controllable in synthetic polymers which can be modified to incorporate adhesion peptides or release biological molecules (272).

In the hydrogel approach, cells are encapsulated in the scaffold during synthesis of the gel which allows homogenous seeding. Hydrogels have also been shown to induce cell maturation and differentiation which makes them an attractive system for basic studies of cardiac development and potential for the delivery of therapeutics to the heart (273, 274). Suspension of CDCs in a hydrogel formed from serum and the glycosoaminoglycan hyaluronic acid, increased the oxygen consumption rate from that of cells in suspension and increased cell retention after transplantation *in vivo* (275). Porous scaffolds can be generated by freeze-drying suspensions poured in molds (267, 276). This type of manufacturing gives flexibility in shape and composition, but limits cell seeding efficacy as most seeded cells remain attached to the scaffold surface (267). Fibrous scaffolds can be manufactured from a large variety of materials by electrospinning which gives control over the nano-scale structure and mechanical properties of the scaffold, but again limits the seeding efficiency (277). Zhang et al. found that, compared to 2D hESC monolayers, hESC-CM in a 3D fibrin-based cardiac patch exhibited significantly higher conduction velocities, longer sarcomeres and enhanced expression of genes involved in cardiac contractile function (244). Potentially the “ultimate scaffold material” is that of decellularized heart tissue since it has the potential to give rise to appropriately structured scaffolds for organ replacement (278).

One noteworthy 3D construct is the engineered heart tissue (EHT) which is a three-dimensional, hydrogel-based muscle construct that is restrained between posts, thereby allowing the cells to contract against a force. EHTs can be generated from isolated heart cells from adult hearts, such as chicken (259) and rat (258) as well as from hESC (279) and hiPSC (280). iPSC-CM EHTs have showed to increase mitochondrial mass, DNA content, and protein abundance (proteome) compared to their 2D counterparts. Moreover they were found to generate more energy via oxidation of glucose, lactate, and fatty acid with a decreased reliance on anaerobic glycolysis, generating 2.3-fold more ATP by oxidation than 2D hiPSC-CMs (245). Mills et al. have developed a technique for growing 3D cardiac organoids in 96 well plates (281). The cells form dense muscle bundles in serum-free conditions developed to promote metabolic and proliferative maturation. They found that addition of palmitate increased the force of contraction and expression of ventricular myosin light chain 2. Interestingly, addition of insulin promoted cell cycling and so was not included in the maturation medium comprising 1 mM glucose and 0.1 mM palmitate. By comparing contractile properties of organoids grown in maturation medium with those in control medium, they found that maturation medium reduced activation time and relaxation time, thereby recapitulating changes seen during human cardiomyocytes development.

Thus, three-dimensional culture conditions have induced binucleation, rod-like cell shape, increased sarcomere alignment, more mature electrophysiology, and calcium handling properties (247). Complex, multi-cellular cell sheets are now being developed from mixtures of iPSC-CMs with fibroblasts and endothelial cells (282) or with mesenchymal cells (283) which exhibit more mature physiology or drug responses but these are still grown in non-physiologic culture media (282, 283). What is needed now, is routine modulation of the culture medium, as discussed above, to cells in 3D constructs to fully induce metabolic maturation.

## Conclusions

The field of CPC biology has come a long way over the last 20 years. Beating sheets of cardiomyocytes can now be generated routinely from ESCs and iPSCs in large numbers. However, the maturity of the cardiomyocytes remains a cause for concern if they are to be used to validate new drug compounds, detect cardiotoxicity or recapitulate the cardiac physiology of patients with genetic disorders. Long term culture can aid maturation, as can culture in 3D constructs, but a fully mature cardiomyocytes should express the appropriate level of substrate transporters and mitochondrial proteins. Further work is required to determine appropriate cell culture conditions to enable pluripotent stem cell-derived cardiomyocytes to achieve the metabolic flexibility of the adult heart.

## Author contributions

All authors contributed conception and design for the manuscript. SM-M and CL wrote the first draft of the manuscript. All authors wrote sections of the manuscript and contributed to manuscript revision, read and approved the submitted version. SM-M and CL are joint first authors.

### Conflict of interest statement

The authors declare that the research was conducted in the absence of any commercial or financial relationships that could be construed as a potential conflict of interest.

## References

[1] Velagaleti RS Pencina MJ Murabito JM Wang TJ Parikh NI D'Agostino RB. Long-term trends in the incidence of heart failure after myocardial infarction. Circulation (2008)118:2057–62. 10.1161/CIRCULATIONAHA.108.784215 18955667 PMC2729712

[2] Go AS Mozaffarian D Roger VL Benjamin EJ Berry JD Blaha MJ Heart disease and stroke statistics−2014 update: a report from the american heart association. Circulation (2014) 129:e28–292. 10.1161/01.cir.0000441139.02102.80 24352519 PMC5408159

[3] Fuster V. Global burden of cardiovascular disease: time to implement feasible strategies and to monitor results. J Am Coll Cardiol. (2014)64:520–2. 10.1016/j.jacc.2014.06.1151 25082587

[4] Goldfinger JZ Adler ED. End-of-life options for patients with advanced heart failure. Curr Heart Fail Rep. (2010)7:140–7. 10.1007/s11897-010-0017-5 20585999

[5] Kumar A Cannon CP Acute coronary syndromes: diagnosis and management, part I. Mayo Clin Proc. (2009)84:917–38. 10.4065/84.10.917 19797781 PMC2755812

[6] Kumar A Cannon CP. Acute coronary syndromes: diagnosis and management, part II. Mayo Clin Proc. (2009)84:1021–36. 10.1016/S0025-6196(11)60674-5 19880693 PMC2770915

[7] Kirklin JK Naftel DC Pagani FD Kormos RL Stevenson LW Blume ED. Sixth INTERMACS annual report: a 10,000-patient database. J Hear Lung Transplant. (2014)33:555–64. 10.1016/j.healun.2014.04.010 24856259

[8] Perricone AJ Vander Heide RS. Novel therapeutic strategies for ischemic heart disease. Pharmacol Res. (2014)89:36–45. 10.1016/j.phrs.2014.08.004 25193582 PMC4182165

[9] Toyoda Y Guy TS Kashem A. Present status and future perspectives of heart transplantation. Circ J. (2013)77:1097–110. 10.1253/circj.CJ-13-0296 23614963

[10] Laflamme MA Murry CE. Heart regeneration. Nature (2011)473:326–35. 10.1038/nature10147 21593865 PMC4091722

[11] Bergmann O Bhardwaj RD Bernard S Zdunek S Barnabé-Heider F Walsh S. Evidence for cardiomyocyte renewal in humans. Science (2009)324:98–102. 10.1126/science.1164680 19342590 PMC2991140

[12] Gersh BJ Simari RD Behfar A Terzic CM Terzic A. Cardiac cell repair therapy: a clinical perspective. Mayo Clin Proc. (2009)84:876–92. 10.4065/84.10.876 19797777 PMC2755807

[13] Vincent SD Buckingham ME. How to make a heart. The origin and regulation of cardiac progenitor cells. Curr Top Dev Biol. (2010)90:1–41. 10.1016/S0070-2153(10)90001-X 20691846

[14] Lalit PA Hei DJ Raval AN Kamp TJ. Induced pluripotent stem cells for post-myocardial infarction repair: remarkable opportunities and challenges. Circ Res. (2014)114:1328–45. 10.1161/CIRCRESAHA.114.300556 24723658 PMC4016859

[15] Bruyneel AAN Sehgal A Malandraki-Miller S Carr C Stem cell therapy for the heart: blind alley or magic bullet? J Cardiovasc Transl Res. (2016)9:405–18. 10.1007/s12265-016-9708-y 27542008 PMC5153828

[16] Tomita S Li RK Weisel RD Mickle DA Kim EJ Sakai T. Autologous transplantation of bone marrow cells improves damaged heart function. Circulation (1999) 100(19 Suppl.):II247–56. 10.1161/01.CIR.100.suppl_2.II-247 10567312

[17] Makino S Fukuda K Miyoshi S Konishi F Kodama H Pan J. Cardiomyocytes can be generated from marrow stromal cells *in vitro*. J Clin Invest. (1999)103:697–705. 10.1172/JCI5298 10074487 PMC408125

[18] Orlic D Kajstura J Chimenti S Jakoniuk I Anderson SM Li B. Bone marrow cells regenerate infarcted myocardium. Nature (2001)410:701–5. 10.1038/35070587 11287958

[19] Beltrami AP Barlucchi L Torella D Baker M Limana F Chimenti S. Adult cardiac stem cells are multipotent and support myocardial regeneration. Cell (2003)114:763–76. 10.1016/S0092-8674(03)00687-1 14505575

[20] Oh H Bradfute SB Gallardo TD Nakamura T Gaussin V Mishina Y. Cardiac progenitor cells from adult myocardium: homing, differentiation, and fusion after infarction. Proc Natl Acad Sci USA. (2003)100:12313–8. 10.1073/pnas.2132126100 14530411 PMC218755

[21] Messina E De Angelis L Frati G Morrone S Chimenti S Fiordaliso F. Isolation and expansion of adult cardiac stem cells from human and murine heart. Circ Res. (2004)95:911–21. 10.1161/01.RES.0000147315.71699.51 15472116

[22] Carr C Stuckey DJ Tan JJ Tan SC Gomes RSM Camelliti P. Cardiosphere-derived cells improve function in the infarcted rat heart for at least 16 weeks - an mri study. PLoS ONE (2011) 6:e25669. 10.1371/journal.pone.0025669 22043289 PMC3197153

[23] Smart N Bollini S Dube KN Vieira JM Zhou B Davidson S. *De novo* cardiomyocytes from within the activated adult heart after injury. Nature (2011)474:640–4. 10.1038/nature10188 21654746 PMC3696525

[24] Cai CL Liang X Shi Y Chu PH Pfaff SL Chen J. Isl1 identifies a cardiac progenitor population that proliferates prior to differentiation and contributes a majority of cells to the heart. Dev Cell. (2003)5:877–89. 10.1016/S1534-5807(03)00363-0 14667410 PMC5578462

[25] Zhou B Gise AV Ma Q Rivera-Feliciano J Pu WT. Nkx2-5- and Isl1-expressing cardiac progenitors contribute to proepicardium. Biochem Biophys Res Commun. (2008)375:450–3. 10.1016/j.bbrc.2008.08.044 18722343 PMC2610421

[26] Chong JJH Yang X Don CW Minami E Liu YW Weyers JJ. Human embryonic-stem-cell-derived cardiomyocytes regenerate non-human primate hearts. Nature (2014)510:273–7. 10.1038/nature13233 24776797 PMC4154594

[27] Laflamme MA Chen KY Naumova AV Muskheli V Fugate JA Dupras SK. Cardiomyocytes derived from human embryonic stem cells in pro-survival factors enhance function of infarcted rat hearts. Nat Biotechnol. (2007)25:1015–24. 10.1038/nbt1327 17721512

[28] Fernandes S Naumova AV Zhu WZ Laflamme MA Gold J Murry CE. Human embryonic stem cell-derived cardiomyocytes engraft but do not alter cardiac remodeling after chronic infarction in rats. J Mol Cell Cardiol. (2010)49:941–9. 10.1016/j.yjmcc.2010.09.008 20854826 PMC2992844

[29] Shiba Y Fernandes S Zhu WZ Filice D Muskheli V Kim J. Human ES-cell-derived cardiomyocytes electrically couple and suppress arrhythmias in injured hearts. Nature (2012)489:322–5. 10.1038/nature11317 22864415 PMC3443324

[30] Carpenter L Carr C Yang CTCT Stuckey DJDJ Clarke K Watt SMSM. Efficient differentiation of human induced pluripotent stem cells generates cardiac cells that provide protection following myocardial infarction in the rat. Stem Cells Dev. (2012)21:977–86. 10.1089/scd.2011.0075 22182484 PMC3315757

[31] Kadota S Pabon L Reinecke H Murry CE. *In vivo* maturation of human induced pluripotent stem cell-derived cardiomyocytes in neonatal and adult rat hearts. Stem Cell Rep. (2017)8:278–89. 10.1016/j.stemcr.2016.10.009 28065644 PMC5311430

[32] Murry CE Soonpaa MH Reinecke H Nakajima H Nakajima HO Rubart M Haematopoietic stem cells do not transdifferentiate into cardiac myocytes in myocardial infarcts. Nature (2004)428:664–8. 10.1038/nature02446 15034593

[33] Balsam LB Wagers AJ Christensen JL Kofidis T Weissman IL Robbins RC. Haematopoietic stem cells adopt mature haematopoietic fates in ischaemic myocardium. Nature (2004)428:668–73. 10.1038/nature02460 15034594

[34] Hatzistergos KE Quevedo H Oskouei BN Hu Q Feigenbaum GS Margitich IS. Bone marrow mesenchymal stem cells stimulate cardiac stem cell proliferation and differentiation. Circ Res. (2010)107:913–22. 10.1161/CIRCRESAHA.110.222703 20671238 PMC3408082

[35] Loffredo FS Steinhauser ML Gannon J Lee RT. Bone marrow-derived cell therapy stimulates endogenous cardiomyocyte progenitors and promotes cardiac repair. Cell Stem Cell (2011)8:389–98. 10.1016/j.stem.2011.02.002 21474103 PMC4148018

[36] Abdel-Latif A Bolli R Tleyjeh IM Montori VM Perin EC Hornung CA. Adult bone marrow-derived cells for cardiac repair: a systematic review and meta-analysis. Arch Intern Med. (2007)167:989–97. 10.1001/archinte.167.10.989 17533201

[37] Breymann C Schmidt D Hoerstrup SP. Umbilical cord cells as a source of cardiovascular tissue engineering. Stem Cell Rev. (2006)2:87–92. 10.1007/s12015-006-0014-y 17237546

[38] Fang CH Jin J Joe JH Song YS So BI Lim SM. *In vivo* differentiation of human amniotic epithelial cells into cardiomyocyte-like cells and cell transplantation effect on myocardial infarction in rats: comparison with cord blood and adipose tissue-derived mesenchymal stem cells. Cell Transplant. (2012)21:1687–96. 10.3727/096368912X653039 22776022

[39] Gimble JM Katz AJ Bunnell BA. Adipose-derived stem cells for regenerative medicine. Circ Res. (2007)100:1249–60. 10.1161/01.RES.0000265074.83288.09 17495232 PMC5679280

[40] Li B Zeng Q Wang H Shao S Mao X Zhang F. Adipose tissue stromal cells transplantation in rats of acute myocardial infarction. Coron Artery Dis. (2007)18:221–7. 10.1097/MCA.0b013e32801235da 17429297

[41] Valina C Pinkernell K Song YH Bai X Sadat S Campeau RJ. Intracoronary administration of autologous adipose tissue-derived stem cells improves left ventricular function, perfusion, and remodelling after acute myocardial infarction. Eur Heart J. (2007)28:2667–77. 10.1093/eurheartj/ehm426 17933755

[42] De Coppi P Bartsch G Jr Siddiqui MM Xu T Santos CC Perin L. Isolation of amniotic stem cell lines with potential for therapy. Nat Biotech. (2007)25:100–6. 10.1038/nbt1274 17206138

[43] Fazel S Cimini M Chen L Li S Angoulvant D Fedak P. Cardioprotective c-kit+ cells are from the bone marrow and regulate the myocardial balance of angiogenic cytokines. J Clin Invest. (2006)116:1865–77. 10.1172/JCI27019 16823487 PMC1483161

[44] Uchida S De Gaspari P Kostin S Jenniches K Kilic A Izumiya Y. Sca1-derived cells are a source of myocardial renewal in the murine adult heart. Stem Cell Rep. (2013)1:397–410. 10.1016/j.stemcr.2013.09.004 24286028 PMC3841250

[45] Sandstedt J Jonsson M Lindahl A Jeppsson A Asp J. C-kit+ CD45- Cells found in the adult human heart represent a population of endothelial progenitor cells. Basic Res Cardiol. (2010)105:545–56. 10.1007/s00395-010-0088-1 20119835

[46] Sandstedt J Jonsson M Dellgren G Lindahl A Jeppsson A Asp J. Human C-kit+CD45- cardiac stem cells are heterogeneous and display both cardiac and endothelial commitment by single-cell qPCR analysis. Biochem Biophys Res Commun. (2014)443:234–8. 10.1016/j.bbrc.2013.11.086 24309111

[47] Jesty SA Steffey MA Lee FK Breitbach M Hesse M Reining S c-kit+ precursors support postinfarction myogenesis in the neonatal, but not adult, heart. Proc Natl Acad Sci USA. (2012)109:13380–5. 10.1073/pnas.1208114109 22847442 PMC3421216

[48] Tallini YN Greene KS Craven M Spealman A Breitbach M Smith J. C-Kit expression identifies cardiovascular precursors in the neonatal heart. Proc Natl Acad Sci USA. (2009)106:1808–13. 10.1073/pnas.0808920106 19193854 PMC2644119

[49] Bolli R Chugh AR D'Amario D Loughran JH Stoddard MF Ikram S. Cardiac stem cells in patients with ischaemic cardiomyopathy (SCIPIO): initial results of a randomised phase 1 trial. Lancet (2011)378:1847–57. 10.1016/S0140-6736(11)61590-0 22088800 PMC3614010

[50] The Lancet Editors Expression of concern: the SCIPIO trial. Lancet. (2014) 383:1279 10.1016/S0140-6736(14)60608-5 24725564 PMC5586533

[51] Ellison GM Vicinanza C Smith AJ Aquila I Leone A Waring CD Adult c-kitpos cardiac stem cells are necessary and sufficient for functional cardiac regeneration and repair. Cell (2013)154:827–42. 10.1016/j.cell.2013.07.039 23953114

[52] Molkentin JD Houser SR. Are resident c-Kit+ cardiac stem cells really all that are needed to mend a broken heart? Circ Res. (2013)113:1037–9. 10.1161/CIRCRESAHA.113.302564 24115067

[53] van Berlo JH Kanisicak O Maillet M Vagnozzi RJ Karch J Lin SCJ c-kit+ cells minimally contribute cardiomyocytes to the heart. Nature (2014)509:337–41. 10.1038/nature13309 24805242 PMC4127035

[54] Sultana N Zhang L Yan J Chen J Cai W Razzaque S Resident c-kit(+) cells in the heart are not cardiac stem cells. Nat Commun. (2015) 6:8701 10.1038/ncomms9701 26515110 PMC4846318

[55] Vicinanza C Aquila I Scalise M Cristiano F Marino F Cianflone E Adult cardiac stem cells are multipotent and robustly myogenic: C-kit expression is necessary but not sufficient for their identification. Cell Death Differ. (2017)24:2101–16. 10.1038/cdd.2017.130 28800128 PMC5686347

[56] Gude NA Firouzi F Broughton KM Ilves K Nguyen KP Payne CR. Cardiac c-Kit biology revealed by inducible transgenesis. Circ Res. (2018)123:57–72. 10.1161/CIRCRESAHA.117.311828 29636378 PMC6192707

[57] Wang X Hu Q Nakamura Y Lee J Zhang G From AHL. The role of the sca-1+/CD31- cardiac progenitor cell population in postinfarction left ventricular remodeling. Stem Cells (2006)24:1779–88. 10.1634/stemcells.2005-0386 16614004

[58] Bailey B Fransioli J Gude NA Alvarez R Zhan X Gustafsson AB. Sca-1 knockout impairs myocardial and cardiac progenitor cell function. Circ Res. (2012)111:750–60. 10.1161/CIRCRESAHA.112.274662 22800687 PMC3463406

[59] van Vliet P Roccio M Smits AM van Oorschot AAM Metz CHG van Veen TAB. Progenitor cells isolated from the human heart: a potential cell source for regenerative therapy. Neth Heart J. (2008)16:163–9. 10.1007/BF03086138 18566670 PMC2431168

[60] Noseda M Harada M McSweeney S Leja T Belian E Macaulay I PDGFRα demarcates the cardiogenic and clonogenic Sca-1^+^ stem cell. Cardiovasc Res. (2014) 103:S107 10.1038/ncomms7930

[61] Smith RR Barile L Cho HC Leppo MK Hare JM Messina E. Regenerative potential of cardiosphere-derived cells expanded from percutaneous endomyocardial biopsy specimens. Circulation (2007)115:896–908. 10.1161/CIRCULATIONAHA.106.655209 17283259

[62] Malliaras K Makkar RR Smith RR Cheng K Wu E Bonow RO. Intracoronary cardiosphere-derived cells after myocardial infarction: evidence of therapeutic regeneration in the final 1-year results of the CADUCEUS trial (CArdiosphere-derived aUtologous stem CElls to reverse ventricular dysfunction). J Am Coll Cardiol. (2014)63:110–22. 10.1016/j.jacc.2013.08.724 24036024 PMC3947063

[63] Lin L Bu L Cai CL Zhang X Evans S. Isl1 is upstream of sonic hedgehog in a pathway required for cardiac morphogenesis. Dev Biol. (2006)295:756–63. 10.1016/j.ydbio.2006.03.053 16687132

[64] Wu SM Fujiwara Y Cibulsky SM Clapham DE Lien CL Schultheiss TM. Developmental origin of a bipotential myocardial and smooth muscle cell precursor in the mammalian heart. Cell (2006)127:1137–50. 10.1016/j.cell.2006.10.028 17123591

[65] Tanaka M Chen Z Bartunkova S Yamasaki N Izumo S The cardiac homeobox gene Csx/Nkx2.5 lies genetically upstream of multiple genes essential for heart development. Development (1999)126:1269–80. 10021345 10.1242/dev.126.6.1269

[66] Thomson JA Itskovitz-Eldor J Shapiro SS Waknitz MA Swiergiel JJ Marshall VS. Embryonic stem cell lines derived from human blastocysts. Science (1998)282:1145–7. 10.1126/science.282.5391.1145 9804556

[67] Kehat I Khimovich L Caspi O Gepstein A Shofti R Arbel G. Electromechanical integration of cardiomyocytes derived from human embryonic stem cells. Nat Biotechnol. (2004)22:1282–9. 10.1038/nbt1014 15448703

[68] Xue T Cho HC Akar FG Tsang SY Jones SP Marban E. Functional integration of electrically active cardiac derivatives from genetically engineered human embryonic stem cells with quiescent recipient ventricular cardiomyocytes: insights into the development of cell-based pacemakers. Circulation (2005)111:11–20. 10.1161/01.CIR.0000151313.18547.A2 15611367

[69] Zimmermann WH. Embryonic and embryonic-like stem cells in heart muscle engineering. J Mol Cell Cardiol. (2011)50:320–6. 10.1016/j.yjmcc.2010.10.027 21040727

[70] Takahashi K Tanabe K Ohnuki M Narita M Ichisaka T Tomoda K. Induction of pluripotent stem cells from adult human fibroblasts by defined factors. Cell (2007)131:861–72. 10.1016/j.cell.2007.11.019 18035408

[71] Karakikes I Ameen M Termglinchan V Wu JC. Human induced pluripotent stem cell-derived cardiomyocytes: insights into molecular, cellular, and functional phenotypes. Circ Res. (2015)117:80–8. 10.1161/CIRCRESAHA.117.305365 26089365 PMC4546707

[72] Lan F Lee AS Liang P Sanchez-Freire V Nguyen PK Wang L. Abnormal calcium handling properties underlie familial hypertrophic cardiomyopathy pathology in patient-specific induced pluripotent stem cells. Cell Stem Cell (2013)12:101–13. 10.1016/j.stem.2012.10.010 23290139 PMC3638033

[73] Liang P Lan F Lee AS Gong T Sanchez-Freire V Wang Y. Drug screening using a library of human induced pluripotent stem cell-derived cardiomyocytes reveals disease-specific patterns of cardiotoxicity. Circulation (2013)127:1677–91. 10.1161/CIRCULATIONAHA.113.001883 23519760 PMC3870148

[74] Denning C Borgdorff V Crutchley J Firth KSA George V Kalra S. Cardiomyocytes from human pluripotent stem cells: from laboratory curiosity to industrial biomedical platform. Biochim Biophys Acta Mol Cell Res. (2016)1863:1728–48. 10.1016/j.bbamcr.2015.10.014 26524115 PMC5221745

[75] Veerman CC Kosmidis G Mummery CL Casini S Verkerk AO Bellin M Immaturity of human stem-cell-derived cardiomyocytes in culture: fatal flaw or soluble problem? Stem Cells Dev. (2015)24:1035–52. 10.1089/scd.2014.0533 25583389

[76] Satin J Itzhaki I Rapoport S Schroder EA Izu L Arbel G. Calcium handling in human embryonic stem cell-derived cardiomyocytes. Stem Cells (2008)26:1961–72. 10.1634/stemcells.2007-0591 18483424

[77] Brito-Martins M Harding SE Ali NN. Beta(1)- and Beta(2)-adrenoceptor responses in cardiomyocytes derived from human embryonic stem cells: comparison with failing and non-failing adult human heart. Br J Pharmacol. (2008)153:751–9. 10.1038/sj.bjp.0707619 18193079 PMC2259205

[78] Don CW Murry CE. Improving survival and efficacy of pluripotent stem cell-derived cardiac grafts. J Cell Mol Med. (2013)17:1355–62. 10.1111/jcmm.12147 24118766 PMC4049630

[79] Bing RJ Siegel A Ungar I Gilbert M. Metabolism of the human heart: II. studies on fat, ketone and amino acid metabolism. Am J Med. (1954)16:504–15. 10.1016/0002-9343(54)90365-4 13148192

[80] Neubauer S. The failing heart–an engine out of fuel. N Engl J Med. (2007)356:1140–51. 10.1056/NEJMra063052 17360992

[81] Neely JR Morgan HE. Relationship between carbohydrate and lipid metabolism and the energy balance of heart muscle. Annu Rev Physiol. (1974)36:413–59. 10.1146/annurev.ph.36.030174.002213 19400669

[82] Evans RD Heather LC Metabolic pathways and abnormalities. Surgery (2016) 34:266−72. 10.1016/j.mpsur.2016.03.010

[83] Taegtmeyer H Young ME Lopaschuk GD Abel ED Brunengraber H Darley-Usmar V. Assessing cardiac metabolism. Circ Res. (2016)118:1659–701. 10.1161/RES.0000000000000097 27012580 PMC5130157

[84] Bar-Even A Flamholz A Noor E Milo R. Rethinking glycolysis: on the biochemical logic of metabolic pathways. Nat Chem Biol. (2012)8:509–17. 10.1038/nchembio.971 22596202

[85] Madeira VMC. Overview of mitochondrial bioenergetics. Methods Mol Biol. (2012)810:1–6. 10.1007/978-1-61779-382-0_1 22057557

[86] Lopaschuk G The role of fatty acid oxidation in cardiac ischemia and reperfusion. Adv Stud Med. (2004) 4:S803–7.

[87] Lopaschuk GD Ussher JR Folmes CDL Jaswal JS Stanley WC. Myocardial fatty acid metabolism in health and disease. Physiol Rev. (2010)90:207–58. 10.1152/physrev.00015.2009 20086077

[88] van der Vusse G Glatz J Stam H Reneman R. Fatty acid homeostasis in the normoxic and ischemic heart. Physiol Rev. (1992)72:881–940. 10.1152/physrev.1992.72.4.881 1438581

[89] Cole MA Jamil AHA Heather LC Murray AJ Sutton ER Slingo M On the pivotal role of PPARa in adaptation of the heart to hypoxia and why fat in the diet increases hypoxic injury. FASEB J. (2016)30:2684–97. 10.1096/fj.201500094R 27103577 PMC5072355

[90] Kim JW Tchernyshyov I Semenza GL Dang CV. HIF-1-mediated expression of pyruvate dehydrogenase kinase: a metabolic switch required for cellular adaptation to hypoxia. Cell Metab. (2006)3:177–85. 10.1016/j.cmet.2006.02.002 16517405

[91] Randle PJ Garland PB Hales CN Newsholme EA The glucose fatty-acid cycle its role in insulin sensitivity and the metabolic disturbances of diabetes mellitus. Lancet (1963)281:785–9. 10.1016/S0140-6736(63)91500-9 13990765

[92] Hue L Taegtmeyer H. The Randle cycle revisited: a new head for an old hat. Am J Physiol Endocrinol Metab. (2009) 297:E578–91. 10.1152/ajpendo.00093.2009 19531645 PMC2739696

[93] Lopaschuk GD Jaswal JS. Energy metabolic phenotype of the cardiomyocyte during development, differentiation, and postnatal maturation. J Cardiovasc Pharmacol. (2010)56:130–40. 10.1097/FJC.0b013e3181e74a14 20505524

[94] Lai L Leone TC Zechner C Schaeffer PJ Kelly SM Flanagan DP Transcriptional coactivators PGC-lα and PGC-lβ control overlapping programs required for perinatal maturation of the heart. Genes Dev. (2008)22:1948–61. 10.1101/gad.1661708 18628400 PMC2492740

[95] Kolwicz SC Purohit S Tian R. Cardiac metabolism and its interactions with contraction, growth, and survival of cardiomyocytes. Circ Res. (2013)113:603–16. 10.1161/CIRCRESAHA.113.302095 23948585 PMC3845521

[96] Lopaschuk GD Spafford MA Marsh DR. Glycolysis is predominant source of myocardial ATP production immediately after birth. Am J Physiol. (1991) 261(6 Pt 2):H1698–705. 1750528 10.1152/ajpheart.1991.261.6.H1698

[97] Breckenridge RA Piotrowska I Ng KE Ragan TJ West JA Kotecha S. Hypoxic regulation of hand1 controls the fetal-neonatal switch in cardiac metabolism. PLoS Biol. (2013) 11:e1001666. 10.1371/journal.pbio.1001666 24086110 PMC3782421

[98] Puente BN Kimura W Muralidhar SA Moon J Amatruda JF Phelps KL. The oxygen-rich postnatal environment induces cardiomyocyte cell-cycle arrest through DNA damage response. Cell (2014)157:565–79. 10.1016/j.cell.2014.03.032 24766806 PMC4104514

[99] Mahmoud AI Kocabas F Muralidhar SA Kimura W Koura AS Thet S. Meis1 regulates postnatal cardiomyocyte cell cycle arrest. Nature (2013)497:249–53. 10.1038/nature12054 23594737 PMC4159712

[100] Attardi G Schatz G. Biogenesis of mitochondria. Annu Rev Cell Biol. (1988)4:289–333. 10.1146/annurev.cb.04.110188.001445 2461720

[101] Mayor F Cuezva JM. Hormonal and metabolic changes in the perinatal period. Biol Neonate. (1985)48:185–96. 2998491 10.1159/000242171

[102] Menendez-Montes I Escobar B Palacios B Gómez MJ Izquierdo-Garcia JL Flores L. Myocardial VHL-HIF signaling controls an embryonic metabolic switch essential for cardiac maturation. Dev Cell (2016)39:724–39. 10.1016/j.devcel.2016.11.012 27997827

[103] Lehman JJ Barger PM Kovacs A Saffitz JE Medeiros DM Kelly DP. Peroxisome proliferator-activated receptor gamma coactivator-1 promotes cardiac mitochondrial biogenesis. J Clin Invest. (2000)106:847–56. 10.1172/JCI10268 11018072 PMC517815

[104] Steinmetz M Quentin T Poppe A Paul T Jux C Changes in expression levels of genes involved in fatty acid metabolism: upregulation of all three members of the PPAR family (a,c,d) and the newly described adiponectin receptor 2, but not adiponectin receptor 1 during neonatal cardiac development of the. Basic Res Cardiol. (2005)100:263–69. 10.1007/s00395-005-0520-0 15754086

[105] Virbasius CMA Virbasius JV Scarpulla RC. NRF-1, an activator involved in nuclear-mitochondrial interactions, utilizes a new DNA-binding domain conserved in a family of developmental regulators. Genes Dev. (1993)7:2431–45. 8253388 10.1101/gad.7.12a.2431

[106] Chung S Dzeja PP Faustino RS Perez-Terzic C Behfar A Terzic A. Mitochondrial oxidative metabolism is required for the cardiac differentiation of stem cells. Nat Clin Pr Cardiovasc Med. (2007) 4 (Suppl. 1):S60–7. 10.1038/ncpcardio0766 17230217 PMC3232050

[107] Larsson NG Wang J Wilhelmsson H Oldfors A Rustin P Lewandoski M. Mitochondrial transcription factor A is necessary for mtDNA maintenance and embryogenesis in mice. Nat Genet. (1998)18:231–6. 10.1038/ng0398-231 9500544

[108] Bushdid PB Osinska H Wacław RR Mołkentin JD Yutzey KE. NFATc3 and NFATc4 are required for cardiac development and mitochondrial function. Circ Res. (2003)92:1305–13. 10.1161/01.RES.0000077045.84609.9F 12750314

[109] Wang T McDonald C Petrenko NB Leblanc M Wang T Giguere V. Estrogen-Related Receptor α (ERRα) and ERRγ are essential coordinators of cardiac metabolism and function. Mol Cell Biol. (2015)35:1281–98. 10.1128/MCB.01156-14 25624346 PMC4355525

[110] Alaynick WA Kondo RP Xie W He W Dufour CR Downes M. ERRγ directs and maintains the transition to oxidative metabolism in the postnatal heart. Cell Metab. (2007)6:13–24. 10.1016/j.cmet.2007.06.007 17618853

[111] Lee SH Wolf PL Escudero R Deutsch R Jamieson SW Thistlethwaite PA. Early expression of angiogenesis factors in acute myocardial ischemia and infarction. N Engl J Med. (2000)342:626–33. 10.1056/NEJM200003023420904 10699162

[112] Cross HR Opie LH Radda GK Clarke K. Is a high glycogen content beneficial or detrimental to the ischemic rat heart? A controversy resolved. Circ Res. (1996)78:482–91. 10.1161/01.RES.78.3.482 8593707

[113] Majmundar AJ Wong WJ Simon MC. Hypoxia-inducible factors and the response to hypoxic stress. Mol Cell. (2010)40:294–309. 10.1016/j.molcel.2010.09.022 20965423 PMC3143508

[114] Gustafsson ÅB. Bnip3 as a dual regulator of mitochondrial turnover and cell death in the myocardium. Pediatr Cardiol. (2011)32:267–74. 10.1007/s00246-010-9876-5 21210091 PMC3051075

[115] Semenza GL. Hypoxia-inducible factor 1 and cardiovascular disease. Annu Rev Physiol. (2014)76:39–56. 10.1146/annurev-physiol-021113-170322 23988176 PMC4696033

[116] Heather LC Carr CA Stuckey DJ Pope S Morten KJ Carter EE. Critical role of complex III in the early metabolic changes following myocardial infarction. Cardiovasc Res. (2010)85:127–36. 10.1093/cvr/cvp276 19666902

[117] Dodd MS Atherton HJ Carr CA Stuckey DJ West JA Griffin J. Impaired *in vivo* mitochondrial krebs cycle activity after myocardial infarction assessed using hyperpolarized magnetic resonance spectroscopy. Circ Cardiovasc Imaging (2014)7:895–904. 10.1161/CIRCIMAGING.114.001857 25201905 PMC4450075

[118] Sharov VG Goussev A Lesch M Goldstein S Sabbah HN. Abnormal mitochondrial function in myocardium of dogs with chronic heart failure. J Mol Cell Cardiol. (1998)30:1757–62. 10.1006/jmcc.1998.0739 9769231

[119] Sharov VG Todor AV Silverman N Goldstein S Sabbah HN. Abnormal mitochondrial respiration in failed human myocardium. J Mol Cell Cardiol. (2000)32:2361–7. 10.1006/jmcc.2000.1266 11113011

[120] Gong G Liu J Liang P Guo T Hu Q Ochiai K. Oxidative capacity in failing hearts. Am J Physiol Heart Circ Physiol. (2003) 285:H541–8. 10.1152/ajpheart.01142.2002 12714322

[121] Casademont J Miró O. Electron transport chain defects in heart failure. Heart Fail Rev. (2002)7:131–9. 10.1023/A:1015372407647 11988637

[122] Marin-Garcia J Goldenthal MJ Moe GW. Mitochondrial pathology in cardiac failure. Cardiovasc Res. (2001) 49:17−26. 10.1016/S0008-6363(00)00241-8 11121792

[123] Quigley AF Kapsa RM Esmore D Hale G Byrne E. Mitochondrial respiratory chain activity in idiopathic dilated cardiomyopathy. J Card Fail. (2000)6:47–55. 10.1016/S1071-9164(00)00011-7 10746819

[124] Taegtmeyer H Sen S Vela D. Return to the fetal gene program: a suggested metabolic link to gene expression in the heart. Ann NY Acad Sci. (2010)1188:191–8. 10.1111/j.1749-6632.2009.05100.x 20201903 PMC3625436

[125] Leong HS Grist M Parsons H Wambolt RB Lopaschuk GD Brownsey R. Accelerated rates of glycolysis in the hypertrophied heart: are they a methodological artifact? Am J Physiol Endocrinol Metab. (2002) 282:E1039–45. 10.1152/ajpendo.00507.2001 11934668

[126] Ellen Kreipke R Wang Y Miklas JW Mathieu J Ruohola-Baker H. Metabolic remodeling in early development and cardiomyocyte maturation. Semin Cell Dev Biol. (2016)52:84–92. 10.1016/j.semcdb.2016.02.004 26912118 PMC4820352

[127] Fragasso G. Deranged cardiac metabolism and the pathogenesis of heart failure. Card Fail Rev. (2016)2:8–13. 10.15420/cfr.2016:5:2 28785448 PMC5490933

[128] Marazzi G Rosanio S Caminiti G Dioguardi FS Mercuro G. The role of amino acids in the modulation of cardiac metabolism during ischemia and heart failure. Curr Pharm Des. (2008)14:2592–604. 10.2174/138161208786071227 18991676

[129] Carubelli V Castrini AI Lazzarini V Gheorghiade M Metra M Lombardi C. Amino acids and derivatives, a new treatment of chronic heart failure? Heart Fail Rev. (2014)20:39–51. 10.1007/s10741-014-9436-9 24925377

[130] Wende AR Brahma MK McGinnis GR Young ME. Metabolic origins of heart failure. JACC Basic Translational Sci. (2017)2:297–310. 10.1016/j.jacbts.2016.11.009 28944310 PMC5609457

[131] Lai L Leone TC Keller MP Martin OJ Broman AT Nigro J. Energy metabolic reprogramming in the hypertrophied and early stage failing heart a multisystems approach. Circ Heart Fail. (2014)7:1022–31. 10.1161/CIRCHEARTFAILURE.114.001469 25236884 PMC4241130

[132] Sun H Olson KC Gao C Prosdocimo DA Zhou M Wang Z. Catabolic defect of branched-chain amino acids promotes heart failure. Circulation (2016)133:2038–49. 10.1161/CIRCULATIONAHA.115.020226 27059949 PMC4879058

[133] Wang W Zhang F Xia Y Zhao S Yan W Wang H. Defective branched chain amino acids catabolism contributes to cardiac dysfunction and remodeling following myocardial infarction. Am J Physiol Hear Circ Physiol. (2016) 311:H1160–9. 10.1152/ajpheart.00114.2016 27542406

[134] Li T Zhang Z Kolwicz SC Abell L Roe ND Kim M. Defective branched-chain amino acid catabolism disrupts glucose metabolism and sensitizes the heart to ischemia-reperfusion injury. Cell Metab. (2017)25:374–85. 10.1016/j.cmet.2016.11.005 28178567 PMC5301464

[135] Biala AK Dhingra R Kirshenbaum LA. Mitochondrial dynamics: orchestrating the journey to advanced age. J Mol Cell Cardiol. (2015)83:37–43. 10.1016/j.yjmcc.2015.04.015 25918048

[136] Lesnefsky EJ Gudz TI Moghaddas S Migita CT Ikeda-Saito M Turkaly PJ. Aging decreases electron transport complex III activity in heart interfibrillar mitochondria by alteration of the cytochrome c binding site. J Mol Cell Cardiol. (2001)33:37–47. 10.1006/jmcc.2000.1273 11133221

[137] Johnson MT Mahmood S Patel MS. Intermediary metabolism and energetics during murine early embryogenesis. J Biol Chem. (2003)278:31457–60. 10.1074/jbc.R300002200 12788927

[138] Folmes CDL Dzeja PP Nelson TJ Terzic A. Metabolic plasticity in stem cell homeostasis and differentiation. Cell Stem Cell. (2012)11:596–606. 10.1016/j.stem.2012.10.002 23122287 PMC3593051

[139] Fisher SA Zhang H Doree C Mathur A Martin-Rendon E Stem cell treatment for acute myocardial infarction. Cochrane database Syst Rev. (2015) 9:CD006536. 10.1002/14651858.CD006536.pub4 PMC8572033 26419913

[140] Zhang J Nuebel E Daley GQ Koehler CM Teitell MA. Metabolic regulation in pluripotent stem cells during reprogramming and self-renewal. Cell Stem Cell. (2012)11:589–95. 10.1016/j.stem.2012.10.005 23122286 PMC3492890

[141] Kondoh H Lleonart ME Nakashima Y Yokode M Tanaka M Bernard D. A high glycolytic flux supports the proliferative potential of murine embryonic stem cells. Antioxid Redox Signal. (2006)9:293–9. 10.1089/ars.2007.9.ft-14 17184172

[142] Moussaieff A Rouleau M Kitsberg D Cohen M Levy G Barasch D. Glycolysis-mediated changes in acetyl-coa and histone acetylation control the early differentiation of embryonic stem cells. Cell Metab. (2015)21:392–402. 10.1016/j.cmet.2015.02.002 25738455

[143] Panopoulos AD Yanes O Ruiz S Kida YS Diep D Tautenhahn R. The metabolome of induced pluripotent stem cells reveals metabolic changes occurring in somatic cell reprogramming. Cell Res. (2012)22:168–77. 10.1038/cr.2011.177 22064701 PMC3252494

[144] Folmes CDL Nelson TJ Martinez-Fernandez A Arrell DK Lindor JZ Dzeja PP. Somatic oxidative bioenergetics transitions into pluripotency-dependent glycolysis to facilitate nuclear reprogramming. Cell Metab. (2011)14:264–71. 10.1016/j.cmet.2011.06.011 21803296 PMC3156138

[145] Zhu S Li W Zhou H Wei W Ambasudhan R Lin T. Reprogramming of human primary somatic cells by OCT4 and chemical compounds. Cell Stem Cell (2010)7:651–5. 10.1016/j.stem.2010.11.015 21112560 PMC3812930

[146] Xu X Duan S Yi F Ocampo A Liu GH Izpisua Belmonte JC. Mitochondrial regulation in pluripotent stem cells. Cell Metab. (2013)18:325–32. 10.1016/j.cmet.2013.06.005 23850316

[147] Prieto J León M Ponsoda X Sendra R Bort R Ferrer-Lorente R. Early ERK1/2 activation promotes DRP1-dependent mitochondrial fission necessary for cell reprogramming. Nat Commun. (2016)7:11124. 10.1038/ncomms11124 27030341 PMC4821885

[148] Cho YM Kwon S Pak YK Seol HW Choi YM Park do J. Dynamic changes in mitochondrial biogenesis and antioxidant enzymes during the spontaneous differentiation of human embryonic stem cells. Biochem Biophys Res Commun. (2006)348:1472–8. 10.1016/j.bbrc.2006.08.020 16920071

[149] Inoue SI Noda S Kashima K Nakada K Hayashi JI Miyoshi H Mitochondrial respiration defects modulate differentiation but not proliferation of hematopoietic stem and progenitor cells. FEBS Lett. (2010)584:3402–9. 10.1016/j.febslet.2010.06.036 20600007

[150] Cai CL Molkentin JD. The elusive progenitor cell in cardiac regeneration: slip slidin' away. Circ Res. (2017)120:400–6. 10.1161/CIRCRESAHA.116.309710 28104772 PMC5260844

[151] Li L Xie T. Stem cell niche: structure and function. Annu Rev Cell Dev Biol. (2005)21:605–31. 10.1146/annurev.cellbio.21.012704.131525 16212509

[152] Warburg O Injuring of respiration the origin of cancer cells. Science (1956)123:309–14. 10.1126/science.123.3191.309 13298683

[153] Munyon W Merchant D. The relation between glucose utilization, lactic acid production and utilization and the growth cycle of L strain fibroblasts. Exp Cell Res. (1959)17:490–98. 10.1016/0014-4827(59)90069-2 13672205

[154] Wang T Marquardt C Foker J. Aerobic glycolysis during lymphocyte proliferation. Nature (1976)261:702–5. 10.1038/261702a0 934318

[155] Hedeskov CJ. Early effects of phytohaemagglutinin on glucose metabolism of normal human lymphocytes. Biochem J. (1968)110:373–80. 10.1042/bj1100373 5726214 PMC1187214

[156] Lunt SY Vander Heiden MG. Aerobic glycolysis: meeting the metabolic requirements of cell proliferation. Annu Rev Cell Dev Biol. (2011)27:441–64. 10.1146/annurev-cellbio-092910-154237 21985671

[157] Board M Lopez C van den Bos C Callaghan R Clarke K Carr C. Acetoacetate is a more efficient energy-yielding substrate for human mesenchymal stem cells than glucose and generates fewer reactive oxygen species. Int J Biochem Cell Biol. (2017)88:75–83. 10.1016/j.biocel.2017.05.007 28483672 PMC5497396

[158] Dos Santos F Andrade PZ Boura JS Abecasis MM Da Silva CL Cabral JMS. *Ex vivo* expansion of human mesenchymal stem cells: a more effective cell proliferation kinetics and metabolism under hypoxia. J Cell Physiol. (2010)223:27–35. 10.1002/jcp.21987 20020504

[159] Hosios AM Hecht VC Danai LV Johnson MO Rathmell JC Steinhauser ML. Amino acids rather than glucose account for the majority of cell mass in proliferating mammalian cells. Dev Cell. (2016)36:540–9. 10.1016/j.devcel.2016.02.012 26954548 PMC4766004

[160] Dai DF Danoviz ME Wiczer B Laflamme MA Tian R. Mitochondrial maturation in human pluripotent stem cell derived cardiomyocytes. Stem Cells Int. (2017) 2017:5153625. 10.1155/2017/5153625 28421116 PMC5380852

[161] Birket MJ Casini S Kosmidis G Elliott DA Gerencser AA Baartscheer A. PGC-1α and reactive oxygen species regulate human embryonic stem cell-derived cardiomyocyte function. Stem Cell Rep. (2013)1:560–74. 10.1016/j.stemcr.2013.11.008 24371810 PMC3871390

[162] Wang Z Yang Y Xiang X Zhu Y Men J He M. Estimation of the normal range of blood glucose in rats. Wei Sheng Yan Jiu. (2010)39:133–7, 142. 20459020

[163] Wolfensohn S Lloyd M Handbook of Laboratory Animal Management and Welfare. 3rd ed Oxford: Blackwell Publishing Ltd (2003).

[164] Perbellini F Gomes RSM Vieira S Buchanan D Malandraki-Miller S Bruyneel A Chronic high-fat feeding affects the mesenchymal cell population expanded from adipose tissue but not cardiac atria. Stem Cells Transl Med. (2015)4:1403–14. 10.5966/sctm.2015-0024 26518239 PMC4675502

[165] Abdelmagid SA Clarke SE Nielsen DE Badawi A El-Sohemy A Mutch DM. Comprehensive profiling of plasma fatty acid concentrations in young healthy canadian adults. PLoS ONE (2015) 10:e0116195zzzz. 10.1371/journal.pone.0116195 25675440 PMC4326172

[166] Vander Heiden MG Cantley LC Thompson CB. Understanding the Warburg effect: the metabolic requirements of cell proliferation. Science (2009)324:1029–33. 10.1126/science.1160809 19460998 PMC2849637

[167] Lee YK Ng KM Lai WH Chan YC Lau YM Lian Q. Calcium homeostasis in human induced pluripotent stem cell-derived cardiomyocytes. Stem Cell Rev. (2011)7:976–86. 10.1007/s12015-011-9273-3 21614516 PMC3226695

[168] Kim C Wong J Wen J Wang S Wang C Spiering S. Studying arrhythmogenic right ventricular dysplasia with patient-specific iPSCs. Nature (2013)494:105–10. 10.1038/nature11799 23354045 PMC3753229

[169] Da Rocha AM Campbell K Mironov S Jiang J Mundada L Guerrero-Serna G. HiPSC-CM monolayer maturation state determines drug responsiveness in high throughput pro-arrhythmia screen. Sci Rep. (2017) 7:13834. 10.1038/s41598-017-13590-y 29061979 PMC5653750

[170] Barr RL Lopaschuk GD. Direct measurement of energy metabolism in the isolated working rat heart. J Pharmacol Toxicol Methods (1997)38:11–7. 10.1016/S1056-8719(97)86574-4 9339411

[171] Belke DD Larsen TS Lopaschuk GD Severson DL. Glucose and fatty acid metabolism in the isolated working mouse heart. Am J Physiol. (1999) 277(4 Pt 2):R1210–7. 10.1152/ajpregu.1999.277.4.R1210 10516264

[172] Jang DH Greenwood JC Spyres MB Eckmann DM. Measurement of mitochondrial respiration and motility in acute care. J Intensive Care Med. (2017)32:86–94. 10.1177/0885066616658449 27443317 PMC6902634

[173] Brand MD Nicholls DG. Assessing mitochondrial dysfunction in cells. Biochem J. (2011) 435(Pt 2): 297–312. 10.1042/BJ20110162 21726199 PMC3076726

[174] Heather LC Cole MA Tan JJ Ambrose LJA Pope S Abd-Jamil AH. Metabolic adaptation to chronic hypoxia in cardiac mitochondria. Basic Res Cardiol. (2012)107:268–79. 10.1007/s00395-012-0268-2 22538979

[175] Tan SC Gomes RSM Yeoh KK Perbellini F Malandraki-Miller S Ambrose L. Preconditioning of cardiosphere-derived cells with hypoxia or prolyl-4-hydroxylase inhibitors increases stemness and decreases reliance on oxidative metabolism. Cell Transplant. (2016)25:35–53. 10.3727/096368915X687697 25751158 PMC6042641

[176] Murry CE Field LJ Menasché P. Cell-based cardiac repair reflections at the 10-year point. Circulation (2005)112:3174–83. 10.1161/CIRCULATIONAHA.105.546218 16286608

[177] Barile L Messina E Giacomello A Marbán E. Endogenous cardiac stem cells. Prog Cardiovasc Dis. (2007)50:31–48. 10.1016/j.pcad.2007.03.005 17631436

[178] Smits AM van Vliet P Metz CH Korfage T Sluijter JP Doevendans PA. Human cardiomyocyte progenitor cells differentiate into functional mature cardiomyocytes: an *in vitro* model for studying human cardiac physiology and pathophysiology. Nat Protoc. (2009)4:232–43. 10.1038/nprot.2008.229 19197267

[179] Goumans MJ de Boer TP Smits AM van Laake LW van Vliet P Metz CHG. TGF-β1 induces efficient differentiation of human cardiomyocyte progenitor cells into functional cardiomyocytes *in vitro*. Stem Cell Res. (2008)1:138–49. 10.1016/j.scr.2008.02.003 19383394

[180] Ye J Boyle A Shih H Sievers RE Zhang Y Prasad M. Sca-1 + cardiosphere-derived cells are enriched for isl1-expressing cardiac precursors and improve cardiac function after myocardial injury. PLoS ONE (2012) 7:e30329. 10.1371/journal.pone.0030329 22272337 PMC3260268

[181] Smith AJ Lewis FC Aquila I Waring CD Nocera A Agosti V. Isolation and characterization of resident endogenous c-Kit+ cardiac stem cells from the adult mouse and rat heart. Nat Protoc. (2014)9:1662–81. 10.1038/nprot.2014.113 24945383

[182] Matsuura K Nagai T Nishigaki N Oyama T Nishi J Wada H. Adult cardiac Sca-1-positive cells differentiate into beating cardiomyocytes. J Biol Chem. (2004)279:11384–91. 10.1074/jbc.M310822200 14702342

[183] Oyama T Nagai T Wada H Naito AT Matsuura K Iwanaga K. Cardiac side population cells have a potential to migrate and differentiate into cardiomyocytes *in vitro* and *in vivo*. J Cell Biol. (2007)176:329–41. 10.1083/jcb.200603014 17261849 PMC2063959

[184] Fukuda K. Development of regenerative cardiomyocytes from mesenchymal stem cells for cardiovascular tissue engineering. Artif Organs. (2001)25:187–93. 10.1046/j.1525-1594.2001.025003187.x 11284885

[185] Rangappa S Fen C Lee EH Bongso A Wei ESK. Transformation of adult mesenchymal stem cells isolated from the fatty tissue into cardiomyocytes. Ann Thorac Surg. (2003)75:775–9. 10.1016/S0003-4975(02)04568-X 12645692

[186] Qian Q Qian H Zhang X Zhu W Yan Y Ye S. 5-azacytidine induces cardiac differentiation of human umbilical cord-derived mesenchymal stem cells by activating extracellular regulated kinase. Stem Cells Dev. (2012)21:67–75. 10.1089/scd.2010.0519 21476855 PMC3245671

[187] Xu W Zhang X Qian H Zhu W Sun X Hu J. Mesenchymal stem cells from adult human bone marrow differentiate into a cardiomyocyte phenotype *in vitro*. Exp Biol Med. (2004)229:623–31. 10.1177/153537020422900706 15229356

[188] Naeem N Haneef K Kabir N Iqbal H Jamall S Salim A. DNA methylation inhibitors, 5-azacytidine and zebularine potentiate the transdifferentiation of rat bone marrow mesenchymal stem cells into cardiomyocytes. Cardiovasc Ther. (2013)31:201–9. 10.1111/j.1755-5922.2012.00320.x 22954287

[189] Bearzi C Rota M Hosoda T Tillmanns J Nascimbene A De Angelis A. Human cardiac stem cells. Proc Natl Acad Sci USA. (2007)104:14068–73. 10.1073/pnas.0706760104 17709737 PMC1955818

[190] Linke A Muller P Nurzynska D Casarsa C Torella D Nascimbene A. Stem cells in the dog heart are self-renewing, clonogenic, and multipotent and regenerate infarcted myocardium, improving cardiac function. Proc Natl Acad Sci USA. (2005)102:8966–71. 10.1073/pnas.0502678102 15951423 PMC1157041

[191] Stresemann C Lyko F. Modes of action of the DNA methyltransferase inhibitors azacytidine and decitabine. Int J Cancer (2008)123:8–13. 10.1002/ijc.23607 18425818

[192] Issa JPJ. DNA methylation as a therapeutic target in cancer. Clin Cancer Res. (2007)13:1634–7. 10.1158/1078-0432.CCR-06-2076 17363514

[193] Kaur K Yang J Eisenberg CA Eisenberg LM. 5-Azacytidine promotes the transdifferentiation of cardiac cells to skeletal myocytes. Cell Reprog. (2014)16:1–7. 10.1089/cell.2014.0021 25090621

[194] Wan Safwani WKZ Makpol S Sathapan S Chua KH. 5-Azacytidine is insufficient for cardiogenesis in human adipose-derived stem cells. J Negat Results Biomed. (2012) 11:3. 10.1186/1477-5751-11-3 22221649 PMC3274438

[195] Takahashi T Lord B Schulze PC Fryer RM Sarang SS Gullans SR. Ascorbic acid enhances differentiation of embryonic stem cells into cardiac myocytes. Circulation (2003)107:1912–6. 10.1161/01.CIR.0000064899.53876.A3 12668514

[196] Cao N Liu Z Chen Z Wang J Chen T Zhao X. Ascorbic acid enhances the cardiac differentiation of induced pluripotent stem cells through promoting the proliferation of cardiac progenitor cells. Cell Res. (2012)22:219–36. 10.1038/cr.2011.195 22143566 PMC3351910

[197] Choi KM Seo YK Yoon HH Song KY Kwon SY Lee HS. Effect of ascorbic acid on bone marrow-derived mesenchymal stem cell proliferation and differentiation. J Biosci Bioeng. (2008)105:586–94. 10.1263/jbb.105.586 18640597

[198] Lim JY Kim WH Kim J Park SI. Involvement of TGF-beta1 signaling in cardiomyocyte differentiation from P19CL6 cells. Mol Cells (2007)24:431–6. 18182860

[199] McCulloch CA Tenenbaum HC. Dexamethasone induces proliferation and terminal differentiation of osteogenic cells in tissue culture. Anat Rec. (1986)215:397–402. 10.1002/ar.1092150410 3740474

[200] Jaiswal N Haynesworth SE Caplan AI Bruder SP. Osteogenic differentiation of purified, culture-expanded human mesenchymal stem cells *in vitro*. J Cell Biochem. (1997)64:295–312. 10.1002/(SICI)1097-4644(199702)64:2<295::AID-JCB12>3.0.CO;2-I 9027589

[201] Hamidouche Z Haý E Vaudin P Charbord P Schüle R Marie PJ. FHL2 mediates dexamethasone-induced mesenchymal cell differentiation into osteoblasts by activating Wnt/beta-catenin signaling-dependent Runx2 expression. FASEB J. (2008)22:3813–22. 10.1096/fj.08-106302 18653765

[202] Chang PL Blair HC Zhao X Chien YW Chen D Tilden AB. Comparison of fetal and adult marrow stromal cells in osteogenesis with and without glucocorticoids. Connect Tissue Res. (2006)47:67–76. 10.1080/03008200600584074 16754512

[203] Kattman SJ Witty AD Gagliardi M Dubois NC Niapour M Hotta A. Stage-specific optimization of activin/nodal and BMP signaling promotes cardiac differentiation of mouse and human pluripotent stem cell lines. Cell Stem Cell (2011)8:228–40. 10.1016/j.stem.2010.12.008 21295278

[204] Kehat I Kenyagin-Karsenti D Snir M Segev H Amit M Gepstein A. Human embryonic stem cells can differentiate into myocytes with structural and functional properties of cardiomyocytes. J Clin Invest. (2001)108:407–14. 10.1172/JCI200112131 11489934 PMC209357

[205] Lian X Zhang J Zhu K Kamp TJ Palecek SP Insulin inhibits cardiac mesoderm, not mesendoderm, formation during cardiac differentiation of human pluripotent stem cells and modulation of canonical wnt signaling can rescue this inhibition. Stem Cells (2013)31:447–57. 10.1002/stem.1289 23193013 PMC3582800

[206] Lian X Zhang J Azarin SM Zhu K Hazeltine LB Bao X. Directed cardiomyocyte differentiation from human pluripotent stem cells by modulating Wnt/β-catenin signaling under fully defined conditions. Nat Protoc. (2013)8:162–75. 10.1038/nprot.2012.150 23257984 PMC3612968

[207] Lian X Hsiao C Wilson G Zhu K Hazeltine LB Azarin SM. Robust cardiomyocyte differentiation from human pluripotent stem cells via temporal modulation of canonical Wnt signaling. Proc Natl Acad Sci USA. (2012) 109:E1848–57. 10.1073/pnas.1200250109 22645348 PMC3390875

[208] Paige SL Osugi T Afanasiev OK Pabon L Reinecke H Murry CE. Endogenous wnt/β-Catenin signaling is required for cardiac differentiation in human embryonic stem cells. PLoS ONE (2010) 5:e11134. 10.1371/journal.pone.0011134 20559569 PMC2886114

[209] Mummery CL Zhang J Ng ES Elliott DA Elefanty AG Kamp TJ. Differentiation of human embryonic stem cells and induced pluripotent stem cells to cardiomyocytes: a methods overview. Circ Res. (2012)111:344–58. 10.1161/CIRCRESAHA.110.227512 22821908 PMC3578601

[210] Tzahor E. Wnt/β-Catenin signaling and cardiogenesis: timing does matter. Dev Cell (2007)13:10–3. 10.1016/j.devcel.2007.06.006 17609106

[211] Kwon C Arnold J Hsiao EC Taketo MM Conklin BR Srivastava D. Canonical Wnt signaling is a positive regulator of mammalian cardiac progenitors. Proc Natl Acad Sci USA. (2007)104:10894–9. 10.1073/pnas.0704044104 17576928 PMC1904134

[212] Lin L Cui L Zhou W Dufort D Zhang X Cai CL. beta-Catenin directly regulates Islet1 expression in cardiovascular progenitors and is required for multiple aspects of cardiogenesis. Proc Natl Acad Sci USA. (2007)104:9313–8. 10.1073/pnas.0700923104 17519333 PMC1890491

[213] Ueno S Weidinger G Osugi T Kohn AD Golob JL Pabon L. Biphasic role for Wnt/beta-catenin signaling in cardiac specification in zebrafish and embryonic stem cells. Proc Natl Acad Sci USA. (2007)104:9685–90. 10.1073/pnas.0702859104 17522258 PMC1876428

[214] Nathan E Monovich A Tirosh-Finkel L Harrelson Z Rousso T Rinon A. The contribution of Islet1-expressing splanchnic mesoderm cells to distinct branchiomeric muscles reveals significant heterogeneity in head muscle development. Development (2008)135:647–57. 10.1242/dev.007989 18184728 PMC5851587

[215] Zhang J Wilson GF Soerens AG Koonce CH Yu J Palecek SP. Functional cardiomyocytes derived from human induced pluripotent stem cells. Circ Res. (2009) 104:e30−41. 10.1161/CIRCRESAHA.108.192237 19213953 PMC2741334

[216] Xu C Police S Rao N Carpenter MK. Characterization and enrichment of cardiomyocytes derived from human embryonic stem cells. Circ Res. (2002)91:501–8. 10.1161/01.RES.0000035254.80718.91 12242268

[217] Passier R Oostwaard DW Snapper J Kloots J Hassink RJ Kuijk E. Increased cardiomyocyte differentiation from human embryonic stem cells in serum-free cultures. Stem Cells (2005)23:772–80. 10.1634/stemcells.2004-0184 15917473

[218] Laflamme MA Gold J Xu C Hassanipour M Rosler E Police S. Formation of human myocardium in the rat heart from human embryonic stem cells. Am J Pathol. (2005)167:663–71. 10.1016/S0002-9440(10)62041-X 16127147 PMC1698736

[219] Graichen R Xu X Braam SR Balakrishnan T Norfiza S Sieh S. Enhanced cardiomyogenesis of human embryonic stem cells by a small molecular inhibitor of p38 MAPK. Differentiation (2008)76:357–70. 10.1111/j.1432-0436.2007.00236.x 18021257

[220] Freund C Davis RP Gkatzis K Ward-van Oostwaard D Mummery CL. The first reported generation of human induced pluripotent stem cells (iPS cells) and iPS cell-derived cardiomyocytes in the Netherlands. Neth Heart J. (2010)18:51–4. 20111645 PMC2810037

[221] Yang L Soonpaa MH Adler ED Roepke TK Kattman SJ Kennedy M. Human cardiovascular progenitor cells develop from a KDR+ embryonic-stem-cell-derived population. Nature (2008)453:524–8. 10.1038/nature06894 18432194

[222] Burridge PW Matsa E Shukla P Lin ZC Churko JM Ebert AD. Chemically defined generation of human cardiomyocytes. Nat Methods (2014)11:855–60. 10.1038/nmeth.2999 24930130 PMC4169698

[223] Zhang J Klos M Wilson GF Herman AM Lian X Raval KK. Extracellular matrix promotes highly efficient cardiac differentiation of human pluripotent stem cells: the matrix sandwich method. Circ Res. (2012)111:1125–36. 10.1161/CIRCRESAHA.112.273144 22912385 PMC3482164

[224] Zorzano A Liesa M Palacín M. Role of mitochondrial dynamics proteins in the pathophysiology of obesity and type 2 diabetes. Int J Biochem Cell Biol. (2009)41:1846–54. 10.1016/j.biocel.2009.02.004 19703653

[225] Paltauf-Doburzynska J Malli R Graier WF. Hyperglycemic conditions affect shape and Ca2+ homeostasis of mitochondria in endothelial cells. J Cardiovasc Pharmacol. (2004)44:423–36. 10.1097/01.fjc.0000139449.64337.1b 15454850

[226] Simsek T Kocabas F Zheng J Deberardinis RJ Mahmoud AI Olson EN. The distinct metabolic profile of hematopoietic stem cells reflects their location in a hypoxic niche. Cell Stem Cell (2010)7:380–90. 10.1016/j.stem.2010.07.011 20804973 PMC4159713

[227] Pattappa G Heywood HK de Bruijn JD Lee DA. The metabolism of human mesenchymal stem cells during proliferation and differentiation. J Cell Physiol. (2011)226:2562–70. 10.1002/jcp.22605 21792913

[228] Mathieu J Zhang Z Nelson A Lamba DA Reh TA Ware C. Hypoxia induces re-entry of committed cells into pluripotency. Stem Cell. (2013)31:1737–48. 10.1002/stem.1446 23765801 PMC3921075

[229] Ng KM Lee YK Chan YC Lai WH Fung ML Li RA. Exogenous expression of HIF-1 alpha promotes cardiac differentiation of embryonic stem cells. J Mol Cell Cardiol. (2010)48:1129–37. 10.1016/j.yjmcc.2010.01.015 20116384

[230] Medley TL Furtado M Lam NT Idrizi R Williams D Verma PJ. Effect of oxygen on cardiac differentiation in mouse iPS cells: role of hypoxia inducible factor-1 and Wnt/beta-catenin signaling. PLoS ONE (2013) 8:e80280. 10.1371/journal.pone.0080280 24265804 PMC3827186

[231] Gaber N Gagliardi M Patel P Kinnear C Zhang C Chitayat D. Fetal reprogramming and senescence in hypoplastic left heart syndrome and in human pluripotent stem cells during cardiac differentiation. Am J Pathol. (2013)183:720–34. 10.1016/j.ajpath.2013.05.022 23871585

[232] Fynes K Tostoes R Ruban L Weil B Mason C Veraitch FS. The differential effects of 2% oxygen preconditioning on the subsequent differentiation of mouse and human pluripotent stem cells. Stem Cells Dev. (2014)23:1910–22. 10.1089/scd.2013.0504 24734982

[233] Sauer H Rahimi G Hescheler J Wartenberg M. Role of reactive oxygen species and phosphatidylinositol 3-kinase in cardiomyocyte differentiation of embryonic stem cells. FEBS Lett. (2000)476:218–23. 10.1016/S0014-5793(00)01747-6 10913617

[234] Li J Stouffs M Serrander L Banfi B Bettiol E Charnay Y. The NADPH oxidase NOX4 drives cardiac differentiation: role in regulating cardiac transcription factors and MAP kinase activation. Mol Biol Cell (2006)17:3978–88. 10.1091/mbc.e05-06-0532 16775014 PMC1556380

[235] Crespo FL Sobrado VR Gomez L Cervera AM McCreath KJ. Mitochondrial reactive oxygen species mediate cardiomyocyte formation from embryonic stem cells in high glucose. Stem Cells (2010)28:1132–42. 10.1002/stem.441 20506541

[236] Lundy SD Zhu WZ Regnier M Laflamme MA. Structural and functional maturation of cardiomyocytes derived from human pluripotent stem cells. Stem Cells Dev. (2013)22:1991–2002. 10.1089/scd.2012.0490 23461462 PMC3699903

[237] Chan YC Ting S Lee YK Ng KM Zhang J Chen Z. Electrical stimulation promotes maturation of cardiomyocytes derived from human embryonic stem cells. J Cardiovasc Transl Res. (2013)6:989–99. 10.1007/s12265-013-9510-z 24081385

[238] Ruan JL Tulloch NL Razumova MV Saiget M Muskheli V Pabon L. Mechanical stress conditioning and electrical stimulation promote contractility and force maturation of induced pluripotent stem cell-derived human cardiac tissue. Circulation (2016)134:1557–67. 10.1161/CIRCULATIONAHA.114.014998 27737958 PMC5123912

[239] Yang X Rodriguez M Pabon L Fischer KA Reinecke H Regnier M. Tri-iodo-l-thyronine promotes the maturation of human cardiomyocytes-derived from induced pluripotent stem cells. J Mol Cell Cardiol. (2014)72:296–304. 10.1016/j.yjmcc.2014.04.005 24735830 PMC4041732

[240] Hazeltine LB Simmons CS Salick MR Lian X Badur MG Han W. Effects of substrate mechanics on contractility of cardiomyocytes generated from human pluripotent stem cells. Int J Cell Biol. (2012) 2012:508294. 10.1155/2012/508294 22649451 PMC3357596

[241] Lieu DK Fu J Chiamvimonvat N Tung KWC McNerney GP Huser T. Mechanism-based facilitated maturation of human pluripotent stem cell-derived cardiomyocytes. Circ Arrhythmia Electrophysiol. (2013) 6:191−201. 10.1161/CIRCEP.111.973420 23392582 PMC3757253

[242] Liu J Lieu DK Siu CW Fu JD Tse HF Li RA. Facilitated maturation of Ca2+ handling properties of human embryonic stem cell-derived cardiomyocytes by calsequestrin expression. Am J Physiol Cell Physiol. (2009) 297:C152−9. 10.1152/ajpcell.00060.2009 19357236 PMC2711646

[243] Kuppusamy KT Jones DC Sperber H Madan A Fischer KA Rodriguez ML. Let-7 family of microRNA is required for maturation and adult-like metabolism in stem cell-derived cardiomyocytes. Proc Natl Acad Sci USA. (2015) 112:E2785–94. 10.1073/pnas.1424042112 25964336 PMC4450404

[244] Zhang D Shadrin IY Lam J Xian HQ Snodgrass HR Bursac N. Tissue-engineered cardiac patch for advanced functional maturation of human ESC-derived cardiomyocytes. Biomaterials (2013)34:5813–20. 10.1016/j.biomaterials.2013.04.026 23642535 PMC3660435

[245] Ulmer BM Stoehr A Schulze ML Patel S Gucek M Mannhardt I. Contractile work contributes to maturation of energy metabolism in hipsc-derived cardiomyocytes. Stem Cell Rep. (2018)10:834–47. 10.1016/j.stemcr.2018.01.039 29503093 PMC5919410

[246] Huebsch N Loskill P Deveshwar N Spencer CI Judge LM Mandegar MA. Miniaturized iPS-cell-derived cardiac muscles for physiologically relevant drug response analyses. Sci Rep. (2016) 6:24726. 10.1038/srep24726 27095412 PMC4837370

[247] Besser RR Ishahak M Mayo V Carbonero D Claure I Agarwal A. Engineered microenvironments for maturation of stem cell derived cardiac myocytes. Theranostics (2018)8:124–40. 10.7150/thno.19441 29290797 PMC5743464

[248] Drawnel FM Boccardo S Prummer M Delobel F Graff A Weber M. Disease modeling and phenotypic drug screening for diabetic cardiomyopathy using human induced pluripotent stem cells. Cell Rep. (2014)9:810–20. 10.1016/j.celrep.2014.09.055 25437537

[249] Correia C Koshkin A Duarte P Hu D Teixeira A Domian I. Distinct carbon sources affect structural and functional maturation of cardiomyocytes derived from human pluripotent stem cells. Sci Rep. (2017) 7:8590. 10.1038/s41598-017-08713-4 28819274 PMC5561128

[250] Rana P Anson B Engle S Will Y. Characterization of human-induced pluripotent stem cell-derived cardiomyocytes: bioenergetics and utilization in safety screening. Toxicol Sci. (2012)130:117–31. 10.1093/toxsci/kfs233 22843568

[251] Shimizu T. Fabrication of pulsatile cardiac tissue grafts using a novel 3-dimensional cell sheet manipulation technique and temperature-responsive cell culture surfaces. Circ Res. (2002) 90:40e−48. 10.1161/hh0302.105722 11861428

[252] Carrier RL Papadaki M Rupnick M Schoen FJ Bursac N Langer R. Cardiac tissue engineering: cell seeding, cultivation parameters, and tissue construct characterization. Biotechnol Bioeng. (1999)64:580–9. 10.1002/(SICI)1097-0290(19990905)64:5<580::AID-BIT8>3.0.CO;2-X 10404238

[253] Leor J Aboulafia-Etzion S Dar A Shapiro L Barbash IM Battler A. Bioengineered cardiac grafts: a new approach to repair the infarcted myocardium? Circulation (2000) 102 (19 Suppl. 3):III56–61. 10.1161/01.CIR.102.suppl_3.III-56 11082363

[254] Li RK Jia ZQ Weisel RD Mickle DAG Choi A Yau TM. Survival and function of bioengineered cardiac grafts. Circulation (1999) 100 (Suppl. 2):II-63–II-69. 10567280 10.1161/01.cir.100.suppl_2.ii-63

[255] Bursac N Papadaki M Cohen RJ Schoen FJ Eisenberg SR Carrier R. Cardiac muscle tissue engineering: toward an *in vitro* model for electrophysiological studies. Am J Physiol. (1999) 277:H433–44. 10444466 10.1152/ajpheart.1999.277.2.H433

[256] Kofidis T de Bruin JL Yamane T Tanaka M Lebl DR Swijnenburg RJ. Stimulation of paracrine pathways with growth factors enhances embryonic stem cell engraftment and host-specific differentiation in the heart after ischemic myocardial injury. Circulation (2005)111:2486–93. 10.1161/01.CIR.0000165063.09283.A8 15883216

[257] Van Luyn MJA Tio RA Gallego Y Van Seijen XJ Plantinga JA De Leij LFMH DeJongste MJL. Cardiac tissue engineering: characteristics of in unison contracting two- and three-dimensional neonatal rat ventricle cell (co)-cultures. Biomaterials (2002)23:4793–801. 10.1016/S0142-9612(02)00230-2 12361618

[258] Zimmermann WH Fink C Kralisch D Remmers U Weil J Eschenhagen T. Three-dimensional engineered heart tissue from neonatal rat cardiac myocytes. Biotechnol Bioeng. (2000)68:106–14. 10.1002/(SICI)1097-0290(20000405)68:1<106::AID-BIT13>3.0.CO;2-3 10699878

[259] Eschenhagen T Fink C Remmers U Scholz H Wattchow J Weil J. Three-dimensional reconstitution of embryonic cardiomyocytes in a collagen matrix: a new heart muscle model system. FASEB J. (1997)11:683–94. 10.1096/fasebj.11.8.9240969 9240969

[260] Zimmermann WH Schneiderbanger K Schubert P Didi M Munzel F Heubach JF. Tissue engineering of a differentiated cardiac muscle construct. Circ Res. (2002)90:223–30. 10.1161/hh0202.103644 11834716

[261] Bian W Badie N Himel HD Bursac N. Robust T-tubulation and maturation of cardiomyocytes using tissue-engineered epicardial mimetics. Biomaterials (2014)35:3819–28. 10.1016/j.biomaterials.2014.01.045 24508078 PMC3952205

[262] Zhang W Kong CW Tong MH Chooi WH Huang N Li RA. Maturation of human embryonic stem cell-derived cardiomyocytes (hESC-CMs) in 3D collagen matrix: effects of niche cell supplementation and mechanical stimulation. Acta Biomater. (2017)49:204–17. 10.1016/j.actbio.2016.11.058 27890729

[263] Daley WP Peters SB Larsen M. Extracellular matrix dynamics in development and regenerative medicine. J Cell Sci. (2008) 121(Pt 3):255–64. 10.1242/jcs.006064 18216330

[264] Aszódi A Legate KR Nakchbandi I Fässler R. What mouse mutants teach us about extracellular matrix function. Annu Rev Cell Dev Biol. (2006)22:591–621. 10.1146/annurev.cellbio.22.010305.104258 16824013

[265] Radisic M Park H Shing H Consi T Schoen FJ Langer R. Functional assembly of engineered myocardium by electrical stimulation of cardiac myocytes cultured on scaffolds. Proc Natl Acad Sci USA. (2004)101:18129–34. 10.1073/pnas.0407817101 15604141 PMC539727

[266] Zimmermann WH Melnychenko I Wasmeier G Didie M Naito H Nixdorff U. Engineered heart tissue grafts improve systolic and diastolic function in infarcted rat hearts. Nat Med. (2006)12:452–8. 10.1038/nm1394 16582915

[267] Chen Q Bruyneel A Clarke K Carr C Czernuszka J Collagen-based scaffolds for potential application of heart valve tissue engineering. J Tissue Sci Eng. (2012) S11:3–8. 10.4172/2157-7552.S11-003

[268] Hansen A Eder A Bonstrup M Flato M Mewe M Schaaf S. Development of a drug screening platform based on engineered heart tissue. Circ Res. (2010)107:35–44. 10.1161/CIRCRESAHA.109.211458 20448218

[269] Christman KL Vardanian AJ Fang Q Sievers RE Fok HH Lee RJ. Injectable fibrin scaffold improves cell transplant survival, reduces infarct expansion, and induces neovasculature formation in ischemic myocardium. J Am Coll Cardiol. (2004)44:654–60. 10.1016/j.jacc.2004.04.040 15358036

[270] Khademhosseini A Eng G Yeh J Kucharczyk PA Langer R Vunjak-Novakovic G. Microfluidic patterning for fabrication of contractile cardiac organoids. Biomed Microdevices (2007)9:149–57. 10.1007/s10544-006-9013-7 17146728

[271] Chen Q Bruyneel A Carr C Czernuszka J Bio-mechanical properties of novel bi-layer collagen-elastin scaffolds for heart valve tissue engineering. Procedia Eng. (2013)59:247–54. 10.1016/j.proeng.2013.05.118

[272] Yu J Lee AR Lin WH Lin CW Wu YK Tsai WB. Electrospun PLGA fibers incorporated with functionalized biomolecules for cardiac tissue engineering. Tissue Eng Part A. (2014)20:1896–907. 10.1089/ten.tea.2013.0008 24471778 PMC4086675

[273] Jacot JG Kita-Matsuo H Wei KA Vincent Chen HS Omens JH Mercola M. Cardiac myocyte force development during differentiation and maturation. Ann NY Acad Sci. (2010)1188:121–7. 10.1111/j.1749-6632.2009.05091.x 20201894 PMC2920416

[274] Nachlas ALY Li S Jha R Singh M Xu C Davis ME. Human iPSC-derived mesenchymal stem cells encapsulated in PEGDA hydrogels mature into valve interstitial-like cells. Acta Biomater. (2018)71:235–46. 10.1016/j.actbio.2018.02.025 29505894 PMC5907941

[275] Chan AT Karakas MF Vakrou S Afzal J Rittenbach A Lin X. Hyaluronic acid-serum hydrogels rapidly restore metabolism of encapsulated stem cells and promote engraftment. Biomaterials (2015)73:1–11. 10.1016/j.biomaterials.2015.09.001 26378976 PMC4980097

[276] Ozawa T Mickle DA Weisel RD Koyama N Ozawa S Li RK. Optimal biomaterial for creation of autologous cardiac grafts. Circulation (2002) 106(12 Suppl 1):I176–82. 10.1161/01.cir.0000032901.55215.cc 12354729

[277] Zong X Bien H Chung CY Yin L Fang D Hsiao BS. Electrospun fine-textured scaffolds for heart tissue constructs. Biomaterials (2005)26:5330–8. 10.1016/j.biomaterials.2005.01.052 15814131

[278] Ott HC Matthiesen TS Goh SK Black LD Kren SM Netoff TI. Perfusion-decellularized matrix: using nature's platform to engineer a bioartificial heart. Nat Med. (2008)14:213–21. 10.1038/nm1684 18193059

[279] Schaaf S Shibamiya A Mewe M Eder A Stöhr A Hirt MN. Human engineered heart tissue as a versatile tool in basic research and preclinical toxicology. PLoS ONE (2011) 6:e26397. 10.1371/journal.pone.0026397 22028871 PMC3197640

[280] Weinberger F Breckwoldt K Pecha S Kelly A Geertz B Starbatty J. Cardiac repair in guinea pigs with human engineered heart tissue from induced pluripotent stem cells. Sci Transl Med. (2016) 8:363ra148. 10.1126/scitranslmed.aaf8781 27807283

[281] Mills RJ Titmarsh DM Koenig X Parker BL Ryall JG Quaife-Ryan GA. Functional screening in human cardiac organoids reveals a metabolic mechanism for cardiomyocyte cell cycle arrest. Proc Natl Acad Sci USA. (2017) 114:E8372–81. 10.1073/pnas.1707316114 28916735 PMC5635889

[282] Amano Y Nishiguchi A Matsusaki M Iseoka H Miyagawa S Sawa Y. Development of vascularized iPSC derived 3D-cardiomyocyte tissues by filtration Layer-by-Layer technique and their application for pharmaceutical assays. Acta Biomater. (2016)33:110–21. 10.1016/j.actbio.2016.01.033 26821339

[283] Kawatou M Masumoto H Fukushima H Morinaga G Sakata R Ashihara T. Modelling Torsade de Pointes arrhythmias *in vitro* in 3D human iPS cell-engineered heart tissue. Nat Commun. (2017) 8:1078. 10.1038/s41467-017-01125-y 29057872 PMC5715012

